# Muscle sympathetic nerve activity during pregnancy: A systematic review and meta‐analysis

**DOI:** 10.14814/phy2.15626

**Published:** 2023-03-10

**Authors:** Kelly M. Greenwall, Áine Brislane, Brittany A. Matenchuk, Allison Sivak, Margie H. Davenport, Craig D. Steinback

**Affiliations:** ^1^ Program for Pregnancy and Postpartum Health, Physical Activity and Diabetes Laboratory, Faculty of Physical Education and Recreation, the Women and Children's Health Research Institute, and the Alberta Diabetes Institute University of Alberta, and the University of Alberta Libraries Edmonton Alberta Canada

**Keywords:** blood pressure, cardiovascular health, gestational diabetes, hypertensive disorders, muscle sympathetic nerve activity, pregnancy

## Abstract

We conducted a systematic review and meta‐analysis to quantify the impact of healthy and complex pregnancy on muscle sympathetic nerve activity (MSNA) at rest, and in response to stress. Structured searches of electronic databases were performed until February 23, 2022. All study designs (except reviews) were included: population (pregnant individuals); exposures (healthy and complicated pregnancy with direct measures of MSNA); comparator (individuals who were not pregnant, or with uncomplicated pregnancy); and outcomes (MSNA, BP, and heart rate). Twenty‐seven studies (*N* = 807) were included. MSNA burst frequency was higher in pregnancy (*n* = 201) versus non‐pregnant controls (*n* = 194) (Mean Differences [MD], MD: 10.6 bursts/min; 95% CI: 7.2, 14.0; *I* ^2^ = 72%). Accounting for the normative increase in heart rate with gestation, burst incidence was also higher during pregnancy (Pregnant *N* = 189, non‐pregnant *N* = 173; MD: 11 bpm; 95% CI: 8, 13 bpm; *I* ^2^ = 47%; *p* < 0.0001). Meta‐regression analyses confirmed that although sympathetic burst frequency and incidence are augmented during pregnancy, this was not significantly associated with gestational age. Compared to uncomplicated pregnancy, individuals with obesity, obstructive sleep apnea, and gestational hypertension exhibited sympathetic hyperactivity, while individuals with gestational diabetes mellitus or preeclampsia did not. Uncomplicated pregnancies exhibited a lesser response to head‐up tilt, but an exaggerated sympathetic responsiveness to cold pressor stress compared to non‐pregnant individuals. MSNA is higher in pregnant individuals and further increased with some, but not all pregnancy complications. PROSPERO registration number: CRD42022311590.

**Novelty and relevance**

*What Is New?*

The first systematic review and meta‐analysis on muscle sympathetic nerve (re) activity in uncomplicated and complicated pregnancy.

*What Is Relevant?*

Uncomplicated pregnancy is associated with sympathoexcitation (versus the non‐pregnant state) that is associated with prevailing blood pressure. Blunted baroreflex engagement and neuro‐cardiovascular transduction may stabilize blood pressure. Further augmentation in sympathetic nerve activity is observed in pregnancy complications and may precede diagnosis.

*Clinical/Pathophysiological Implications?*

Understanding sympathetic regulation during pregnancy may help identify those at risk of pregnancy complications, aid treatment, improve pregnancy outcomes, and reduce cardiovascular disease risk.

## 1 INTRODUCTION

Pregnancy is a period of rapid and profound cardiovascular adaptation that is necessary to support a successful pregnancy (Brislane et al., 2021). In healthy pregnancy, blood volume increases by 50% until term which is accompanied by peripheral vasodilation. Resting muscle sympathetic nerve activity (MSNA) has been reported to progressively increase compared to non‐pregnant controls (Charkoudian et al., 2017; Fischer et al., 2004; Greenwood et al., 2001, 2003; Hissen et al., 2017; Jarvis et al., 2012; Okada et al., 2015; Reyes et al., 2018; Reyes, Usselman, Skow, et al., 2018; Usselman, Skow, et al., 2015; Usselman, Wakefield, et al., 2015). It is thought that this increase in MSNA may arise in order to regulate blood pressure in the face of vasodilation (Fu & Levine, 2009). However, as we previously discussed in Reyes, Usselman, Skow, et al. (2018), the findings across studies appear heterogeneous with different research groups reporting variable changes in basal MSNA (Reyes et al., 2020). Further, the increase in sympathetic activity across pregnancy has yet to be systematically quantified.

In non‐pregnant populations, basal MSNA appears to be related endothelial function, arterial stiffness, and the development of overt cardiovascular disease, including chronic hypertension. However, these relationships have been demonstrated in mixed cohorts of participants (males and females). Further, the influence of prevailing MSNA on blood pressure appears less in young females compared to males (Baker et al., 2016). Thus, while the “normal” increase in MSNA reported during healthy pregnancy may or may not have a negative influence on cardiometabolic function, it remains possible that excessive sympathoexcitation may contribute to the development of pregnancy related complications including gestational hypertension, preeclampsia, and gestational diabetes. Therefore, investigating sympathetic regulation during pregnancy may be important for the prediction and understanding of gestational complications.

Although there are correlates between sympathetic hyperactivity and cardiometabolic disorders, (Grassi et al., 2020; Maier et al., 2022) sympathetic cardiovascular responsiveness to stress testing may be of equal, or greater importance (Chida & Steptoe, 2010; Kasagi et al., 1995). In pregnancy, reactivity testing imposes additional demands on an already stressed cardiovascular and sympathetic nervous systems. Dysfunctional MSNA responses to summative stress may be indicative of physiological maladaptation of the neurovascular system during pregnancy and may therefore be of additional clinical importance. Nonetheless, few studies have examined MSNA responses to reactivity testing in pregnancy, nor has there been synthesis of reactivity responses among uncomplicated and complicated pregnancies (e.g., those affected by health complications including gestational diabetes, preeclampsia, and obesity). Therefore, we conducted a systematic review and meta‐analyses to quantify (A) basal MSNA in “healthy” pregnancy compared to the non‐pregnant state and across gestation, (B) the influence of pregnancy complications (including gestational hypertension, preeclampsia, gestational diabetes, obstructive sleep apnea, and obesity) on basal MSNA, and (C) MSNA reactivity to stress testing in normotensive and complicated pregnancies.

## 2 METHODS

We followed the Preferred Reporting Items for Systematic Reviews and Meta‐Analyses (PRISMA) guidelines on systematic reviews and meta‐analysis. The protocol was registered with the International Prospective Register of Systematic Reviews (PROSPERO; Registration no. CRD42022311590).

### 2.1 Information sources

A structured search of electronic databases (MEDLINE, EMBASE, CINAHL, Scopus, Web of Science, Cochrane Library, Trip, and ProQuest Dissertations, and Theses) up to February 28, 2022, was performed by a research librarian (A.S.; see the online supplement for complete search strategies; DOI: 10.6084/m9.figshare.20362170; private link: https://figshare.com/s/72ad3090256b475466d0). We also used manual searches of reference lists of reviews and included papers to identify relevant studies. Authors were contacted when data were missing from primary sources. No language or date restrictions were applied.

### 2.2 Eligibility criteria

The study was guided by the participants, interventions, comparisons, outcomes, and study design (PICOS) framework.

### 2.3 Study design

All study designs were eligible for meta‐analysis except for narrative or systematic reviews, letters, commentaries, and editorials. Where relevant, these types of studies have been incorporated narratively.

### 2.4 Population

The population of interest was pregnant women at any stage of pregnancy.

### 2.5 Intervention/exposure

The exposures of interest included any direct measurement of MSNA in pregnancy at baseline and/or during perturbations (i.e., cold pressor test, isometric handgrip exercises, upright tilts, and lower body negative pressure).

### 2.6 Comparison

Eligible comparators included non‐pregnant controls, as well as comparisons between uncomplicated pregnancies, and those with cardiovascular and metabolic complications.

### 2.7 Outcomes

Eligible outcomes included direct measures of MSNA using microneurography. Secondary outcomes included mean arterial pressure, systolic and diastolic blood pressure, and heart rate.

### 2.8 Study selection

After the removal of duplicates, two reviewers (K.M.G. and C.D.S.) independently assessed the titles and abstracts of articles by Covidence. Studies were selected for full‐text review by at least one reviewer. The full texts of included studies were independently screened by two reviewers (K.M.G. and C.D.S.). Disagreements were resolved through discussion and by decision of a third reviewer where necessary.

### 2.9 Data extraction

Two reviewers independently extracted data in Microsoft Excel. If the study had multiple publications, the most recent or complete publication was selected in data synthesis; however, relevant data from all publications related to each unique study were extracted. In order to prevent “overcounting” of participants, all authors who published multiple studies in the area were contacted to clarify whether participants were included from multiple studies (please see Online Supplement pages 7 and 8 for more details). Study characteristics (e.g., study period, study design, and country), population characteristics (e.g., number of participants, pregnancy related complication, weight, body mass index [BMI], and age, parity), intervention/exposure, and outcomes (MSNA burst frequency, burst incidence, total activity, amplitude) were extracted and recorded in a table. For missing data, attempts were made to contact the first, or corresponding authors for additional information. Data were synthesized narratively if authors were unable to provide additional data. In cases where data were not presented numerically, the online application WebPlotDigitizer was used to extract data from figures (Web Plot Digitizer, V.3.11. Texas, USA).

### 2.10 Quality assessment and certainty assessment

Two reviewers (K.G. and Á.B.) independently assessed the risk of bias of each study that was included. We assessed methodological quality of prospective cohort, retrospective cohort, and cross‐sectional studies by using standardized critical appraisal instruments from the Joanna Briggs Institute (JBI) Critical Appraisal of Evidence Effectiveness tool. JBI checklists were used for each study design to determine the extent to which a study had addressed the possibility of bias in its design, conduct, and analysis. Specifically, all studies were screened for potential sources of bias including inappropriate sampling, flawed measurement of exposure, flawed measurement of outcomes, selective/incomplete outcomes, unidentified confounding factors, and inappropriate statistical analysis. The differences in ratings were resolved through discussion. The overall risk of bias of a study was defined as high risk when more than one third of the factors (cross‐sectional and case control studies: ≥3 of 8 factors: cohort studies: ≥4 of 12 factors) were marked as high risk.

### 2.11 Data synthesis

Reviewer Manager v5.4 (Cochrane Collaboration, Copenhagen, Denmark) was used to conduct the statistical analysis. For continuous outcomes, mean values and their standard deviation (SD) were used in the meta‐analysis. When the reported outcome was analyzed using multiple methods (e.g., total MSNA), data were reported as standard mean difference (SMD). SMD effect sizes (SMD) were calculated in Review Manager v5.4 using Hedges' *g* method (similar to Cohen's *d*). As a general rule, an effect size of 0.2, 0.4, and 0.8 is considered small, moderate, and large, respectively. We calculated 95% CIs using the random‐effects model with inverse variance weighting of pool estimates of the association between MSNA outcomes in pregnancy. Statistical significance was defined as a *p*‐value <0.05. Statistical heterogeneity was assessed using *I* ^2^ statistics. In the case of *I* ^2^ > 50%, heterogeneity was explored further with preplanned sensitivity analysis. A priori‐determined subgroup analysis were conducted, when possible, for pregnancy related complications (gestational hypertension, preeclampsia, gestational diabetes, obesity). Data that could not be transformed to be included in a meta‐analysis, the data were narratively synthesized.

## 3 RESULTS

### 3.1 Study characteristics

**FIGURE 1:**
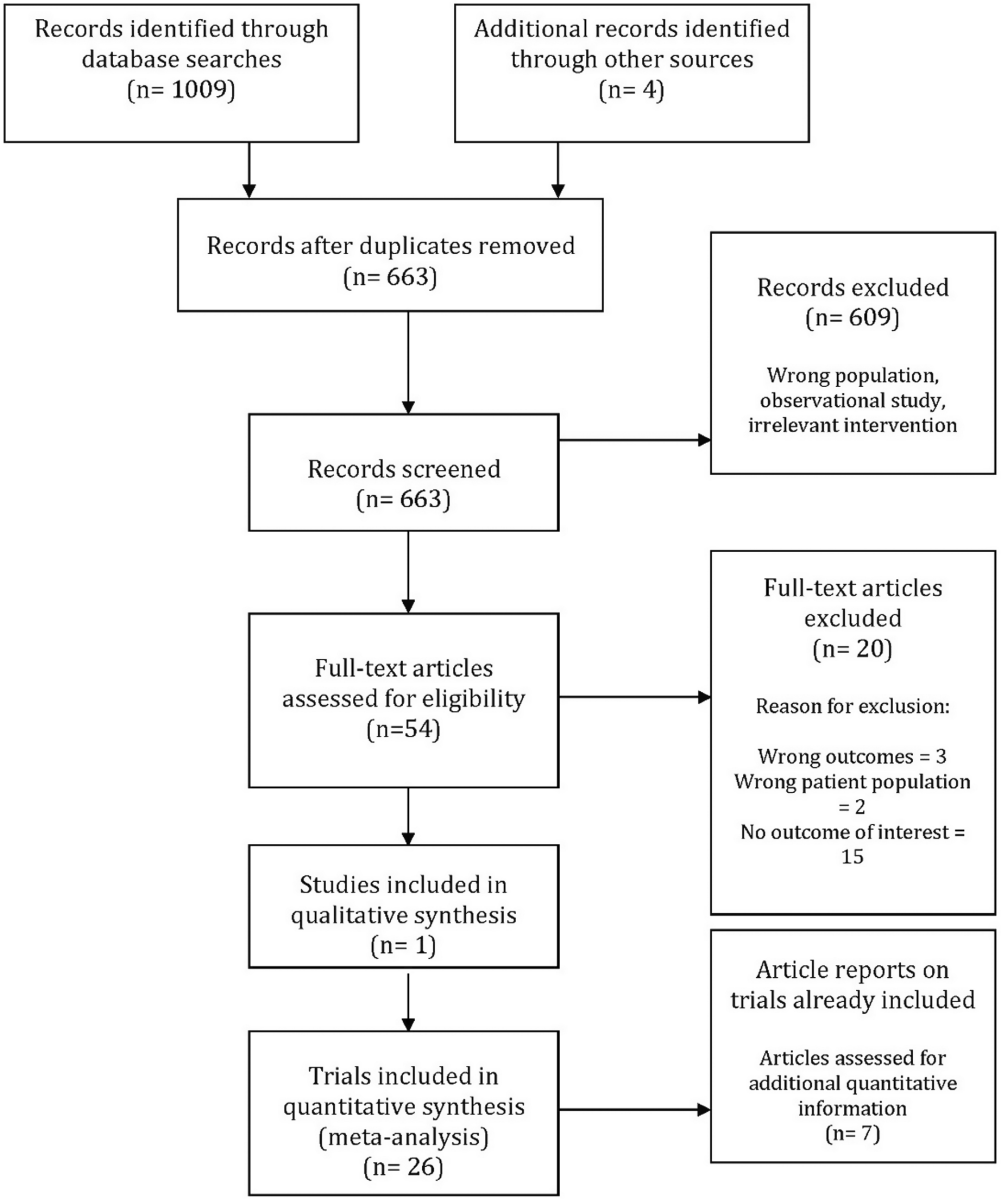
Study flow diagram.

A PRISMA diagram of the study search and selection process is shown in Figure 1. Twenty‐seven studies reporting on 807 participants (663 pregnant) from six countries met the inclusion criteria of the systematic review. Data from these studies were identified from the first, second, or third trimesters of pregnancy. Pregnancy complications included individuals diagnosed with hypertensive disorders of pregnancy (preeclampsia, gestational hypertension, gestational diabetes, obesity, and obstructive sleep apnea) (OSA, Table S1; DOI: 10.6084/m9.figshare.20362170). Gestational hypertension is de novo hypertension (>140/90 mmHg no earlier than 20 weeks gestation) while preeclampsia is similarly diagnosed plus the presence of proteinuria, or evidence of end‐organ dysfunction (e.g., thrombocytopenia, renal insufficiency, impaired liver function, pulmonary edema, and cerebral/visual symptoms). The majority of studies combined both nulliparous and multiparous women (or did not clearly report on parity). Only six studies included exclusively nulliparous (Greenwood et al., 1998, 2001, 2003; Hissen et al., 2017) or multiparous women (Fischer et al., 2004; Stickford et al., 2015).

Five papers reported that at least one participant within control/healthy groups had a personal history of a prior complicated pregnancy. The control group in Reyes et al. (2021) was matched to the group diagnosed with preeclampsia by co‐morbidities (e.g., obesity and GDM). In Fischer et al. (2004), all women had a history of preeclampsia. Badrov et al. (2019) included a low‐risk group of individuals with no history of preeclampsia and a high risk group that had a personal history of preeclampsia. Skow et al. (2020) reported one person with a history of gestational hypertension, and Skow et al. (2021) reported one person with a personal history of GDM and gestational hypertension each.

The studies reporting on individuals with obesity were based on either preconception or early pregnancy BMI. Studies reporting on individuals with OSA were diagnosed with the condition during pregnancy.

Three corresponding authors were sent emails requesting additional information or clarification of data. Two responded to the emails, and one provided additional information (see the online supplement for additional information; DOI: 10.6084/m9.figshare.20362170). Some studies included within the review reported on the same participants across multiple studies. In these cases, steps were taken to prevent overcounting of participants (Reyes et al., 2021). Steps included contacting authors to provide specific information related to overlapping data; the paper reporting on the most number of participants was selected and others removed, or in cases of partial overlap of data, this was mathematically accounted for in the total number of participants (described in detail in the online supplement DOI: 10.6084/m9.figshare.20362170). The majority of papers arise from Dr. Qi Fu's research group, as well as our research group. Our team was able to identify and adjust numbers based on on our own data, and Dr. Qi Fu generously provided detailed information about overlap across studies from their lab.

### 3.2 Quality assessment (risk of bias)

JBI tools for observational studies were used to assess the quality of each individual study (see Tables S2–S5; DOI: 10.6084/m9.figshare.20362170). Common sources of bias included lack clarity regarding the validity and reliability of measuring MSNA, lack of confounding factors identified and inability to determine whether appropriate statistical analysis had been used

### 3.3 Basal sympathetic activity in uncomplicated pregnancy

**FIGURE 2:**
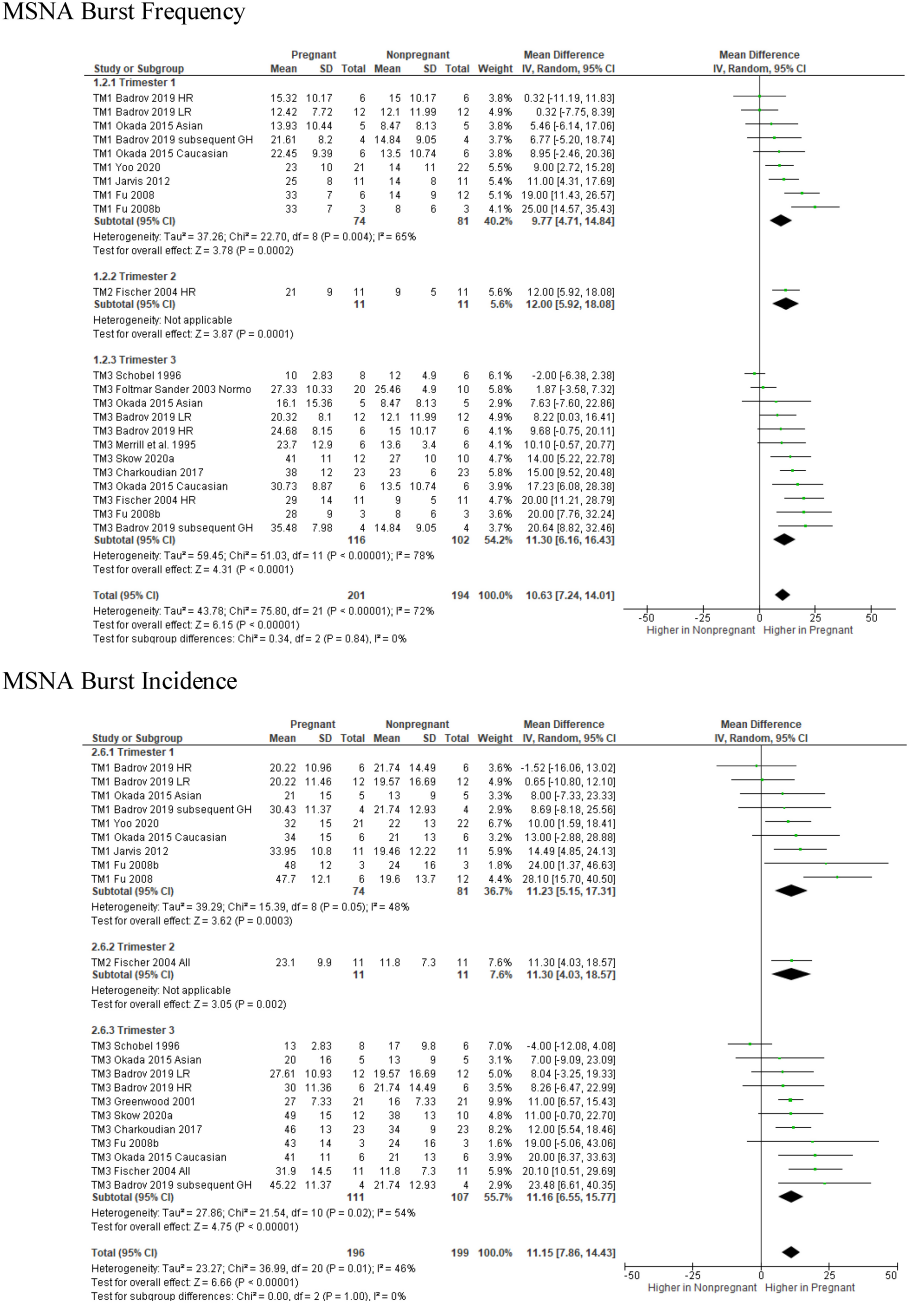
Basal MSNA burst frequency (bursts per minute; top) and incidence (bursts/100 heart beats; bottom) across trimesters during uncomplicated pregnancy. Mean differences are in relation to nonpregnant control groups. MD, mean difference; df, degrees of freedom; GH, subsequently developed gestational hypertension; HR, high risk; IV, inverse variance; LR, low risk.

Overall, baseline MSNA burst frequency was higher in pregnant (*N* = 201) compared with non‐pregnant individuals (*N* = 194; MD: 10.6 bursts/min; 95% CI: 7.2, 14.0; *I* ^2^ = 72%; *p* < 0.0001; see Figure 2). This was apparent as early as trimester 1, and subgroup analyses did not identify any difference between trimesters (χ^2^ = 0.34, *p* = 0.84, *I* ^2^ = 0%) (Figure 2). This pattern of elevated burst frequency in pregnancy is reaffirmed by three longitudinal case studies (reported in two papers) that could not be included in the meta‐analysis (Hissen et al., 2017; Reyes, Usselman, Skow, et al., 2018).

To account for the gestationally dependent change in resting heart rate (pregnant *N* = 189, non‐pregnant *N* = 173; MD: 11 bpm; 95% CI: 8, 13; *I* ^2^ = 47%; *p* < 0.0001; see Figure S1; DOI: 10.6084/m9.figshare.20362170), we analyzed sympathetic burst incidence (bursts/100 heart beats). In this analysis, sympathetic activity remained significantly higher in pregnancy (*N* = 196) compared with the non‐pregnant state (*N* = 199; MD: 11.2 bursts/100 hb; 95% CI: 7.9, 14.4; *I* ^2^ = 64%, *p* < 0.0001; see Figure 2). As with burst frequency, subgroup analyses indicated significant increases in burst incidence as early as the first trimester, but subgroup analyses indicated no differences between trimesters 1, 2, or 3 for basal sympathetic burst incidence (χ^2^ = 0, *I* ^2^ = 0%, *p* = 1.0) (Figure 2).

Meta‐regression analyses confirmed that although sympathetic burst frequency and incidence are augmented during pregnancy, this was not significantly related to gestational age (see Figures S2 and S3; DOI: 10.6084/m9.figshare.20362170).

Few studies reported total MSNA activity, but these limited data also indicated higher sympathetic activity during pregnancy (*N* = 119) compared to non‐pregnant individuals (*N* = 118; SMD: 0.8 AU/min; 95% CI: 0.5, 1.7; *I* ^2^ = 30%; *p* < 0.001 large effect size). This was observed in both trimester 1 (SMD: 0.6 AU/min; 95% CI: 0.2, 0.9; χ^2^ = 4.06, *I* ^2^ = 75.4%, *p* = 0.002; moderate effect size) and trimester 3 (SMD: 1.2 AU/min; 95% CI: 0.7, 1.7, *p* < 0.001; see Figure S4; DOI: 10.6084/m9.figshare.20362170).

**FIGURE 3:**
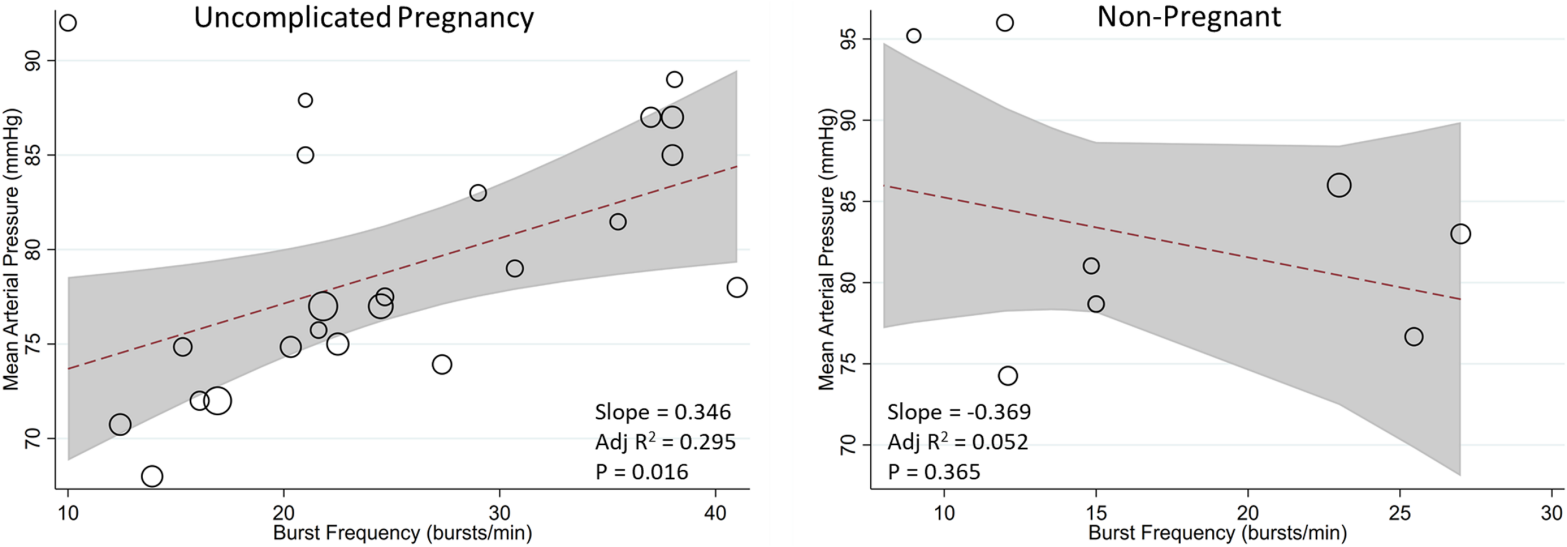
Relationship between MSNA burst frequency and mean arterial pressure in uncomplicated pregnancy (left) and nonpregnant controls (right). A significant relationship between MSNA and mean pressure was observed in uncomplicated pregnancy but not nonpregnant individuals. Weighted meta‐regressions are denoted by the dashed line, with 95% confidence intervals indicated by the shaded region. The size of each dot represents the weight of the particular study within the regression analysis.

Despite clearly higher sympathetic activity, our analyses indicated modest reductions in mean blood pressure during trimester 1 (pregnant *N* = 22, non‐pregnant *N* = 22; MD: −4 mmHg; 95% CI: −8, 0; *I* ^2^ = 0%; *p* = 0.05), trimester 2 (pregnant *N* = 11, non‐pregnant *N* = 11; MD: −12 mmHg; 95% CI: −23, −2; *I* ^2^ = N/A; *p* = 0.02), and trimester 3 (pregnant *N* = 124, non‐pregnant *N* = 107; MD: −3 mmHg; 95% CI: −5, −1; *I* ^2^ = 12%, *p* = 0.01). However, there were no statistical differences between time points for mean blood pressure (χ^2^ = 2.9; *I* ^2^ = 31%; *p =* 0.23; see Figure S5; DOI: 10.6084/m9.figshare.20362170). Overall systolic and diastolic blood pressure was reduced by ~3 mmHg during pregnancy (see Figures S6 and S7; DOI: 10.6084/m9.figshare.20362170). Although there was a modest change in mean arterial pressure, meta‐regression identified that this was related to basal SNA burst frequency during uncomplicated pregnancy, but not in non‐pregnant individuals (Figure 3).

### 3.4 Basal sympathetic activity in complicated pregnancy

**FIGURE 4:**
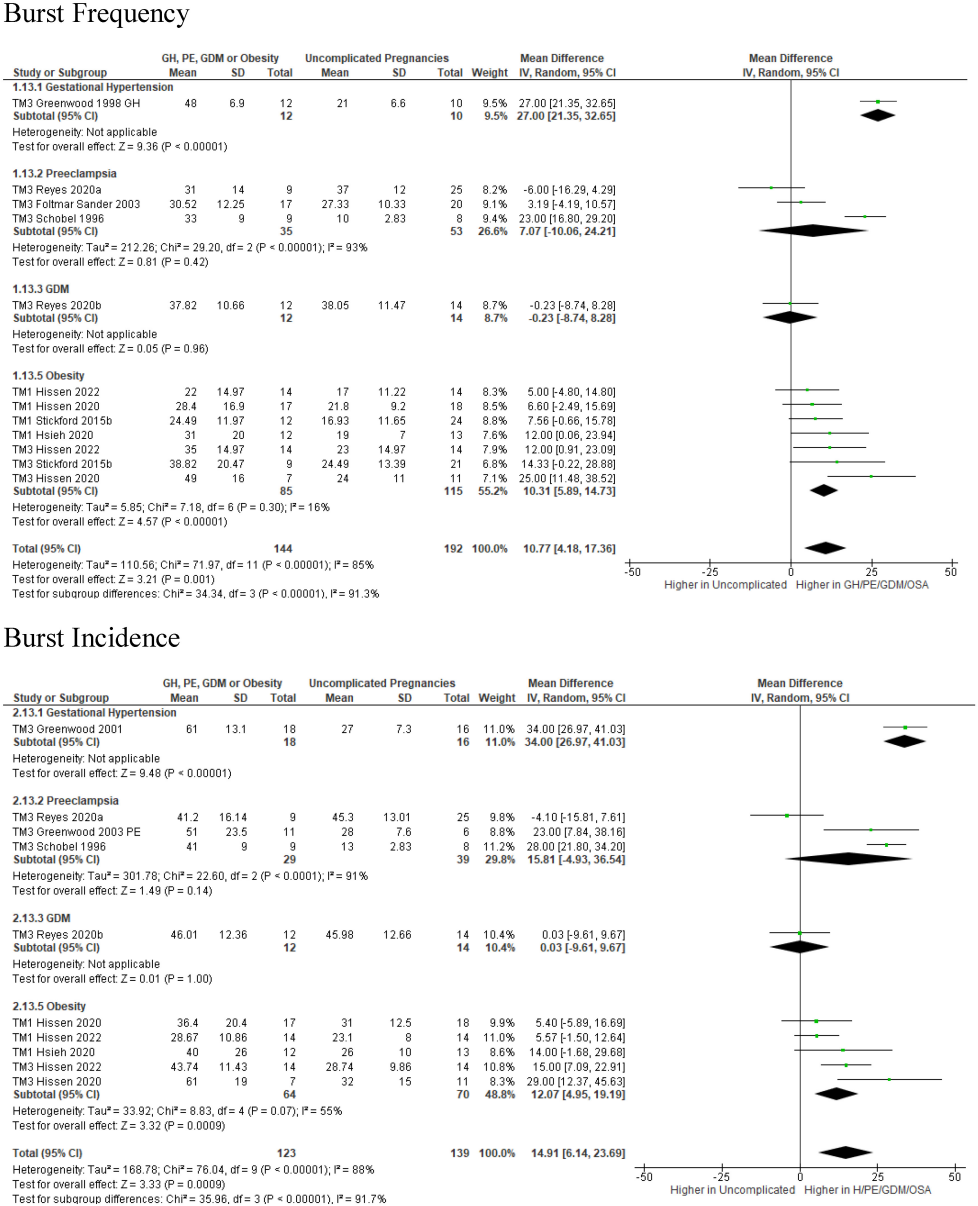
Basal MSNA burst frequency (bursts per minute; top) and incidence (bursts/100 heart beats; bottom) in uncomplicated and complicated pregnancies. df, degrees of freedom;GDM, gestational diabetes mellitus; IV, inverse variance; PE, preeclampsia. Greenwood (2001) utilized old terminology (pregnancy induced hypertension) that falls within the current definition of gestational hypertension.

When considering pregnancy complications as a whole, baseline MSNA burst frequency is higher for complicated (*N* = 144), compared with uncomplicated pregnancies (*N* = 192; MD: 10.8 bursts/min; 95% CI: 4.2, 17.4; *I* ^2^ = 85%; *p* < 0.0001; see Figure 4). When pregnancy complications were evaluated independently, gestational hypertension and obesity exhibited augmented sympathetic activity compared to uncomplicated controls. However, a single study on gestational diabetes indicated no difference in burst frequency versus uncomplicated pregnancy. Contrary to expectation, burst frequency was not higher in preeclampsia (*N* = 35) compared to uncomplicated pregnancy (*N* = 53; MD: 7.07 bursts/min; 95% CI: −10.1, 24.2; *I* ^2^ = 93%; *p* = 0.42). Further, Schobel et al. (1996) was deemed an outlier within this subgroup (i.e., the lowest CI for this study did not cross the highest CI for the other studies). Following a sensitivity analysis removing Schobel et al. (1996) from the analysis, there was no difference between groups (*n* = 71; MD −0.68 burst/min; 95% CI: −9.57, 8.22; see Figure S8; DOI: 10.6084/m9.figshare.2036217; private link: https://figshare.com/s/72ad3090256b475466d0).

A similar pattern and interpretation were observed for burst incidence where pregnancy complications as a whole (*N* = 123) had higher burst compared with uncomplicated pregnancies (*N* = 144; MD: 14.9 bursts/100 hb; 95% CI: 6.1, 23.7; *I* ^2^ = 88%; *p* < 0.0001; see Figure 4). When evaluated independently, burst incidence was higher with gestational hypertension and obesity. Patients with gestational diabetes and preeclampsia had similar burst incidence as uncomplicated pregnancy. Limited data also reported that pregnant women with obesity and obstructive sleep apnea exhibit higher SNA that those obesity alone (see Figures S9 and S10; DOI: 10.6084/m9.figshare.20362170).

As expected, hypertensive pregnancies (gestational hypertension, preeclampsia) exhibited higher blood pressures compared to normotensive pregnant women. Patients with obesity exhibited higher systolic pressure and heart rates compared to normal weight pregnant women (see Figures S11–S14; DOI: 10.6084/m9.figshare.20362170).

### 3.5 Sympathetic reactivity to stress in uncomplicated and complicated pregnancy

#### 3.5.1 Baroreflex stress

**FIGURE 5:**
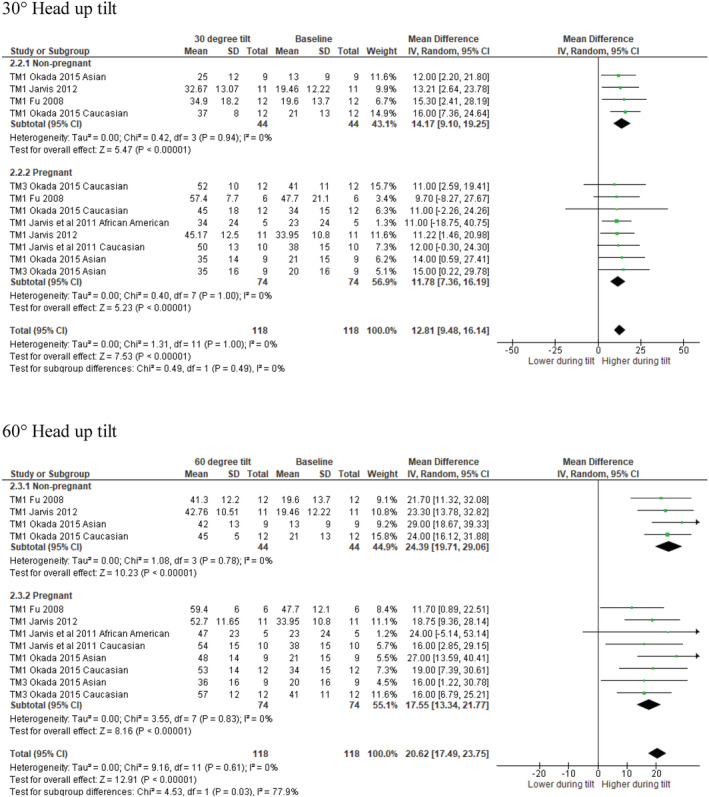
MSNA burst incidence in response to 30 °head‐up tilt (top) and 60° head‐up tilt (bottom) in uncomplicated pregnant and nonpregnant women. MD values are in units per minute. df, degrees of freedom; IV, inverse variance; TM1, trimester 1; TM3, trimester 3.

Studies were identified that evaluated sympathetic responses to sympathetic baroreflex engagement using various degrees of head‐up tilt (30°, 3 studies; 60°, 4 studies); lower body negative pressure and Valsalva's maneuver. MSNA burst frequency (bursts per minute) was elevated in response to 30° and 60° upright tilting to a similar degree in both uncomplicated pregnant and non‐pregnant individuals (see Figures S15 and S16; DOI: 10.6084/m9.figshare.20362170). When accounting for concurrent cardiovagal baroreflex engagement (an increase in heart rate), the change in burst incidence from supine to 30° upright tilting was not different between groups. However, pregnant individuals exhibited a lesser increase in burst incidence compared to non‐pregnant individuals at 60° upright tilting (*p* = 0.03 for subgroup differences, see Figure 5). Stickford et al. (2015) could not be incorporated within our meta‐analysis; however, they demonstrated that women with a history of preeclampsia had a blunted sympathetic response to 60° upright tilting (Δ15 ± 7 bursts/min) compared to women without a history of preeclampsia (Δ26 ± 9 bursts; *p* = 0.014).

One study could not be meta‐analyzed as only change scores were reported. Merill et al. (1995) used −20 mmHg lower body negative pressure as a modest baroreflex stressor. They observed a ∆8.9 ± 13 bursts per minute increase in burst frequency in third trimester pregnant participants (*n* = 6) versus a ∆8.8 ± 3.2 bursts per minute increase in non‐pregnant participants (*n* = 6; *p* = 0.99). These data align with that observed in upright tilt studies.

Schobel et al. (1996) used a 15 s Valsalva maneuver (expiratory pressure of 40 mmHg) to assess baroreflex engagement and found no difference in the sympathetic burst frequency response in normotensive pregnant (*n* = 8, ∆11 ± 6 bursts/min), normotensive non‐pregnant (*n* = 6, ∆12 ± 7 bursts/min), and in women with preeclampsia (*n* = 9, ∆9 ± 6 bursts/min).

Consistent across studies and baroreflex, interventions were similar blood pressure and heart rate responses between pregnant and non‐pregnant women (Figures S17–S24; DOI: 10.6084/m9.figshare.20362170).

#### 3.5.2 Cold pressor test

**FIGURE 6:**
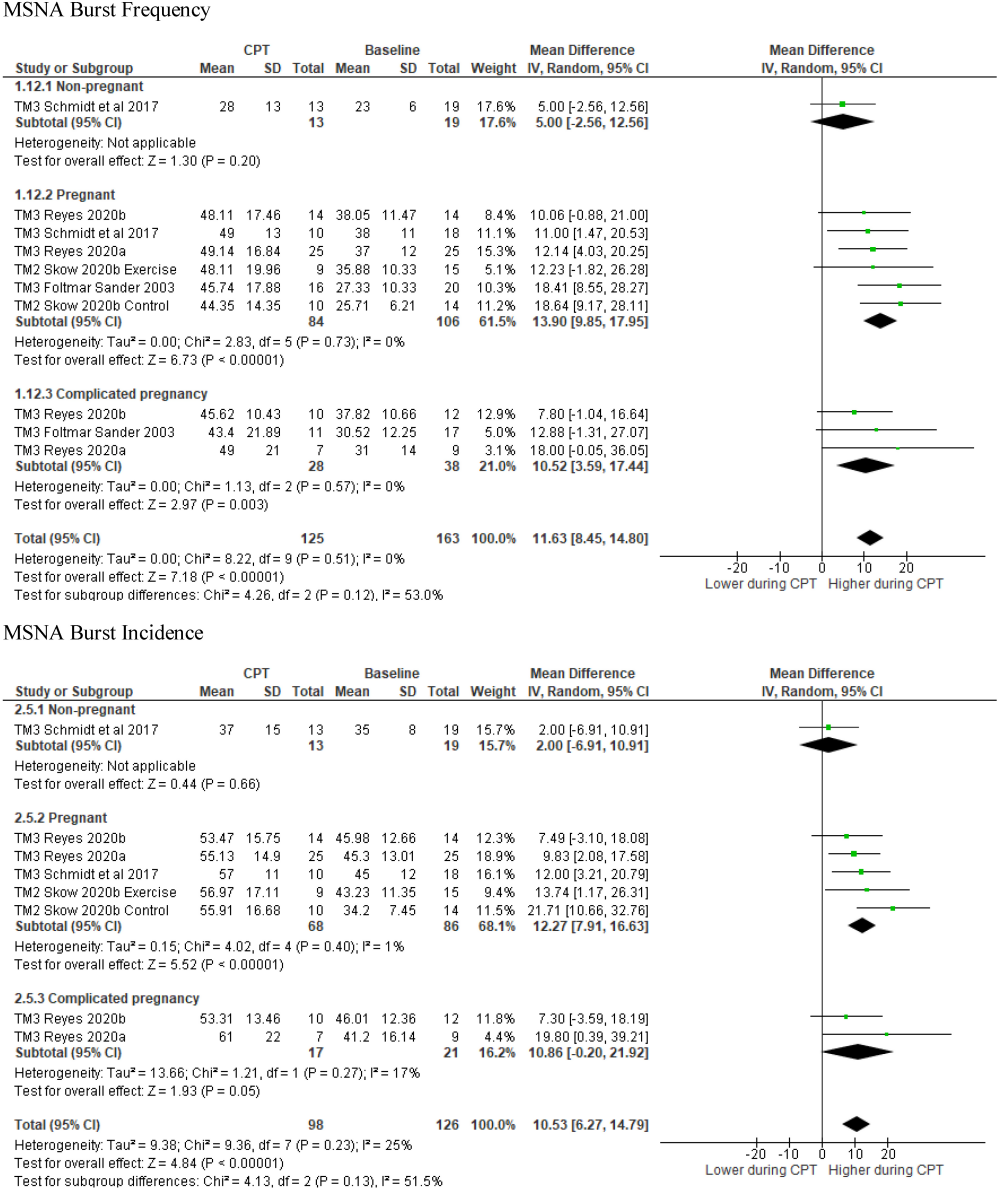
MSNA burst frequency (Top) and incidence (bottom) in response to cold pressor test in nonpregnant, uncomplicated pregnant, and complicated pregnant women. MD values are in units per minute. df, degrees of freedom; GDM, gestational diabetes mellitus; IV, inverse variance; TM1, trimester 1; TM3, trimester 3; PE, preeclampsia.

Our analyses indicated that the increase in Burst frequency during the CPT was greater in uncomplicated pregnancies (*N* = 84; MD: 13.9 bursts/min; 95% CI: 9.8, 18.0; *I* ^2^ = 0%; *p* < 0.0001) versus a limited sample of non‐pregnant individuals (*N* = 13; MD: 5.0 bursts/min; 95% CI: −2.6, 12.6; *p* = 0.20) (subgroup analysis: χ^2^ = 4.1, *I* ^2^ = 75.8%; *p* = 0.04, Figure 6). The MSNA burst frequency response to the CPT was similar between women with complicated pregnancies (including GDM and PE) (*n* = 28; MD: 10.5 bursts/min; 95% CI: 3.6, 17.4; *I* ^2^ = 0%; *p* = 0.003) and those with uncomplicated pregnancies (*N* = 84; MD: 13.9 bursts/min; 95% CI: 9.8, 18.0; *I* ^2^ = 0%; *p* < 0.0001); (Group comparison: χ^2^ = 0.68; *I* ^2^ = 0%; *p* = 0.41). This result remained the same when only women with preeclampsia were considered in the “complicated pregnancy” group. One study could not be included in the meta‐analysis. Schobel et al. reported a similar increase in burst frequency in normotensive pregnant (*n* = 8, Δ 9 ± 8 bursts/min) and pregnant women with preeclampsia (*n* = 9, Δ 9 ± 9 bursts/min) women (Schobel et al., 1996).

A similar outcome was observed for burst incidence, which was unchanged in non‐pregnant individuals (*N* = 13; MD: 2.0 bursts/100 hb; 95% CI: −6.9, 10.9; *p* = 0.66), but increased in uncomplicated pregnancies (*N* = 68; MD: 12.3 bursts/100 hb; 95% CI: 7.9, 16.6; *I* ^2^ = 1%; *p* < 0.0001), with significant differences between the two groups (subgroup analysis: χ^2^ = 4.12, *I* ^2^ = 75.7%; *p* = 0.04; Figure 6). Similarly, the MSNA burst incidence in response to the CPT in women with complicated pregnancies (*N* = 17; MD: 10.9 bursts/100 hb; 95% CI: −0.2, 21.9; *I* ^2^ = 17%; *p* = 0.05) was similar to responses in uncomplicated pregnancies (*N* = 68; MD: 12.3 bursts/100 hb; 95% CI: 7.9, 16.6; *I* ^2^ = 1%; *p* < 0.0001) (Group comparison: χ^2^ = 0.05; *I* ^2^ = 0%; *p* = 0.82). One study could not be included in the meta‐analysis. Greenwood et al. (1998) also reported a Δ 20.4 ± 23 bursts/min increase in burst frequency in normotensive pregnant women (*n* = 11) and a Δ 37.6 ± 29 burst/min increase in women with gestational hypertension (*p* = 0.140).

The total MSNA activity response appeared similar between uncomplicated pregnancy (*N* = 47; SMD: 0.89 AU/min; 95% CI: 0.38, 1.39; *I* ^2^ = 33%; *p* = 0.0007; large effect size) and non‐pregnant individuals (*N* = 13; SMD: 0.60 AU/min; 95% CI: −0.12, 1.32; *p* = 0.10; moderate effect size) (χ^2^ = 0.4; *I* ^2^ = 0%; *p =* 0.43; see Figure S25; DOI: 10.6084/m9.figshare.20362170).

Three other studies included information on CPT responsiveness which we were unable to include in our analyses. Foltmar‐Sander (2003) suggested no difference in the sympathetic responses to CPT during or following pregnancy (data not reported). Schobel et al. (1996) also reported a similar increase in burst frequency in normotensive pregnant (*n* = 8, Δ9 ± 8 bursts/min) and normotensive non‐pregnant (*n* = 6, Δ13 ± 7 bursts/min) women. Greenwood et al. (1998) reported a Δ20.4 ± 23 bursts/min increase in burst frequency in normotensive pregnant women (*n* = 11) with no non‐pregnant comparator group.

Across all studies, there were no differences in the cardiovascular responses to CPT between complicated pregnancy, uncomplicated pregnant and non‐pregnant individuals (see Figures S26–S29; DOI: 10.6084/m9.figshare.20362170).

#### 3.5.3 Acute exercise stress

Only one study compared responses to isometric hang‐grip and postexercise circulatory occlusion between non‐pregnant and normotensive pregnant women (Skow et al., 2020). This precluded the meaningful meta‐analyses. However, this study reported no differences in the burst frequency or burst incidence responses to isometric hand grip (IHG) and post exercise circulatory occlusion (PECO) in pregnant and non‐pregnant women. There were also no differences in cardiovascular response to IHG and PECO between groups (see Figures S30–S37; DOI: 10.6084/m9.figshare.20362170).

Data from one study reported no difference in the change in MSNA burst frequency during IHG for individuals with gestational hypertension (*N* = 13; Δ 37.8 ± 44 bursts/min) versus those with uncomplicated pregnancies (*N* = 11; Δ 23.6 ± 36.8 bursts/min; *p* = 0.425).

#### 3.5.4 Chemoreflex deactivation

In response to hyperoxia, burst frequency was reduced in complicated pregnancies (*N* = 19; MD: −9.60 bursts/min, 95% CI: −17.2, −2.01; *I* ^2^ = 0%; *p* = 0.01). Although hyperoxia did not reduce burst frequency in uncomplicated pregnancies (*N* = 42; MD: −2.45 bursts/min, 95% CI: 2.82, −7.72; *I* ^2^ = 0%; *p* = 0.83), there were no apparent differences between groups (χ^2^ = 2.3; *I* ^2^ = 56.5%; *p* = 0.13; see Figure S38; DOI: 10.6084/m9.figshare.20362170). The MSNA burst incidence response during hyperoxia exhibited a similar pattern, with no differences in responsiveness between groups (χ^2^ = 2.4; *I* ^2^ = 59%; *p* = 0.12; see Figure S9; DOI: 10.6084/m9.figshare.20362170).

There were no differences in cardiovascular responses to the hyperoxia in complicated pregnancies compared to uncomplicated pregnancies (see Figures S40–S43; DOI: 10.6084/m9.figshare.20362170).

## 4 DISCUSSION

In this systematic review and meta‐analyses, we aimed to quantify basal MSNA in uncomplicated and complicated pregnancies compared to the non‐pregnant state. We also sought to quantify MSNA reactivity to stress testing in both uncomplicated and complicated pregnancies. The key finding of these analyses were as follows:

1. Clear sympathoexcitation during uncomplicated pregnancy, manifesting within the first trimester.
2. Pregnancies complicated by gestational hypertension and obesity (but not preeclampsia or gestational diabetes) exhibit augmented sympathetic activity compared to their peers with uncomplicated pregnancies.
3. Pregnant women having uncomplicated pregnancies appear to have a lesser response to head‐up tilt but an exaggerated sympathetic responsiveness to cold pressor stress.

### 4.1 A normative increase in sympathetic activity during pregnancy

Our analyses quantify the normative increase in SNA observed in healthy pregnancy during each trimester, equating to roughly +75%, +135%, and +65% increases sympathetic burst frequency in trimesters 1, 2, and 3, respectively. Further, our meta‐regression demonstrated that changes in sympathetic activity were not associated with gestational age. Looking to those studies that include longitudinal data across trimesters, there is also significant heterogeneity in the time course of sympathetic activation across pregnancy. Additional studies using longitudinal designs are needed, particularly including prepregnant and postpartum assessments to better characterize the trajectory of sympathetic activity with advancing gestation. We also note that three case studies and one cross‐sectional study, including 11 pregnant individuals and a non‐pregnant control group (matched by BMI, age), reported basal MSNA during the second trimester. It is also worth noting that the cohort study by Fischer et al. (2004) reporting data during the second trimester enrolled high‐risk women with a history of preeclampsia, who therefore may not strictly represent “normal” pregnancy. Thus, additional data are critical to evaluate sympathetic activity during the often cited, nadir in arterial blood pressure during this time‐period to understand the contribution of MSNA to this pregnancy adaptation.

Due to the concurrent gestationally dependent increases in heart rate, the reporting both burst frequency as an indicator of the kinetic influence of the sympathetic nervous system and burst incidence as a marker of descending neural drive is critical during pregnancy. That said, we demonstrate augmented burst incidence during healthy pregnancy. This augmentation emphasizes an elevated descending sympathetic drive that is independent of concurrent increases in heart rate. However, these data suffer the same limitations noted above. It is worth highlighting the known limitations of comparing total sympathetic activity (which incorporates burst amplitude) between participants and studies. These include differing recording amplitudes due to variations in proximity to active neurons and differences in normalization procedures between laboratory groups. Nonetheless, normalization of burst amplitudes (e.g., largest burst = 100%) allows for comparison of relative burst amplitude distribution (and total activity) between groups within a given study. Further, the calculation of standardized mean difference for these data accounts for variation in normalization and data presentation between laboratory groups.

### 4.2 The influence of pregnancy complications on sympathetic activity during pregnancy

According to our analysis, basal MSNA is elevated in gestational hypertension, obesity and those with combined obesity and obstructive sleep apnea. This augmentation aligns with that observed in non‐pregnant populations with similar disorders (i.e., hypertension, obesity, and obstructive sleep apnea). While these data can be interpreted as a simple “overlaying” of conditions, Badrov et al. (2019) recently reported that augmented MSNA in the first trimester precedes the development of gestational hypertension. This suggests that augmented sympathoexcitation may be causal in this disorder. In light of this, the adaptation of the sympathetic nervous system across pregnancy may be clinically relevant in identifying those at risk of pregnancy related complications and its management may help reduce this risk. The observation that obesity was related to augmented MSNA in pregnant women suggests that management of weight and weight gain prior to conception and during pregnancy may be important for cardiovascular health. An unexpected finding was that preeclampsia was not associated with augmented sympathetic activity compared to uncomplicated controls. Schobel et al. (1996) were the first to suggest that basal MSNA is higher in women with preeclampsia versus those with a normotensive pregnancy; however, subsequent data that nearly quadruple the included sample size do not replicate this result. It is worth highlighting that Schobel et al. (1996) did not identify a difference in sympathetic activity between normotensive pregnant and non‐pregnant women. In fact, the authors documented a lower MSNA in third trimester women compared to non‐pregnant controls. It is therefore possible that this discrepancy manifested as an apparently supra‐elevated MSNA in women with preeclampsia. Considering that gestational hypertension and preeclampsia are distinct pathologies, it may not be surprising that the involvement of the sympathetic nervous system may differ in these disorders. We found a single study investigating sympathetic regulation in gestational diabetes that indicating no difference in basal MSNA compared to uncomplicated peers. In light of the observed influence of obesity on MSNA during pregnancy noted above, further research is warranted to examine sympathetic regulation during metabolic disorders of pregnancy. Overall, our analyses highlights a significant lack of data from all complicated pregnancies and as such, a huge knowledge gap in this area of research.

### 4.3 Implications for blood pressure control

While a review of the mechanisms underlying the increase in MSNA during pregnancy is beyond the scope of this investigation, one hypothesis is that this increase manifests to counteract a fall in vascular resistance and to maintain stable basal blood pressures. Indeed, our meta‐regression demonstrated a strong relationship between augmented MSNA burst frequency and mean arterial pressure, that is a higher basal MSNA was associated with a higher basal MAP. However, this remains independent of reflex changes in MSNA due to alterations in blood pressure via the baroreflex or vascular responses to fluctuations in MSNA (transduction). Although there were no differences in the sympathetic response to low levels of head‐up tilt (30°), pregnant women exhibited a blunted burst incidence response at 60°, indicative of a lesser baroreflex engagement. This aligns with data that indicate a reduction in sympathetic baroreflex gain during human pregnancy (Usselman, Skow, et al., 2015). Despite augmented basal sympathetic activity a lesser reflex engagement of MSNA during orthostatic stress may be one mechanism contributing to the greater incidence of presyncope/syncope during pregnancy which Gibson et al. estimate to be approximately 30% of pregnant women (Gibson, 2001). When considering reflex sympathetic activation via other stressors such as the cold pressor test, a non‐specific nociceptive stressor, MSNA burst frequency and incidence were greater in pregnant compared with nonpregnant individuals. This occurred without appreciable cardiovascular responses, namely blood pressure, indicating a reduced neurovascular transduction in pregnancy. This is consistent with data from specific studies that indicate a reduced SNA:cardiovascular outcome ratio and blunted neurovascular transduction calculated from signal averaging approaches. Although concurrent changes in input (baroreflex) and output (transduction) gain may serve to attempt to stabilize blood pressure control across gestation, baroreflex dysfunction and or altered transduction may play a role in augmented sympathetic activity and development of pregnancy complications. We have recently reviewed mechanisms that may contribute to altered sympathetic outflow and vascular reactivity, including augmented sex hormones, altered central processing and descending drive, decreased receptor sensitivity or reduced concentrations of sympathetic neurotransmitters (Brislane et al., 2022; Reyes, Usselman, Davenport, & Steinback, 2018). We also refer the reader to a comprehensive review of alterations in baroreflex signaling and brainstem integration which may underly the observed reduction in baroreflex‐mediated MSNA activation during the third trimester of pregnancy (Brooks et al., 2020). Further research is needed to direct linkages between these potential mechanism and functional blood pressure control (presyncope–hypertension) during pregnancy.

### 4.4 Remaining gaps within the literature

A key consideration is how differences in sympathetic activity and reactivity may influence cardiovascular function and health during pregnancy. There are currently little data reported during the second trimester, and a lack of benchmark data on reflex sympathetic activation and control from normotensive pregnancy. Despite the obvious clinical relevance, there was scant research on reflex sympathetic control (particularly baroreflex regulation) in complicated pregnancies. The most general limitation within the current literature is the lack of data from this critically important life stage.

It is also important to note that the pregnancy literature suffers from some of the same limitations as general physiology literature. Importantly, the majority (but not all) data have been reported from Caucasian women. This severely limits the extrapolation of findings between populations. Important, but limited data have started to address this knowledge gap, suggesting that ethnicity (Asian American, African American populations), may be an important underlying modulator of MSNA during pregnancy.

## 5 PERSPECTIVES

This systematic review and meta‐analysis quantified the elevated basal MSNA in uncomplicated pregnancy and identified a relationship between sympathetic activity and mean blood pressure. Women with an uncomplicated pregnancy exhibit a reduced baroreflex responsiveness to head‐up tilt, and a similar cardiovascular response despite augmented sympathetic responsiveness, to the cold pressor test (indicative of reduced neuro‐cardiovascular transduction) compared to nonpregnant women. Both are clinically relevant for the control of blood pressure. We demonstrated that gestational hypertension and obesity, but not preeclampsia nor gestational diabetes, appear associated with exacerbated MSNA. We also identified important gaps within the literature related to understanding longitudinal changes in sympathetic regulation during pregnancy. This includes additional investigations related to reflex control during uncomplicated and complicated pregnancies, and improved ethnic diversity. The outcomes from our analyses should provide quantitative benchmarks for field of research.

## FUNDING INFORMATION

This research has been funded by the Natural Sciences and Engineering Research Council of Canada (NSERC; CDS RGPIN‐2020‐05385; MHD RGPIN‐2019‐07219). C.D. Steinback is funded by a HSFC Joint National and Alberta New Investigator Award (HSFC NNIA Steinback). M.H. Davenport is supported by the Christenson Professorship in Healthy Active Living and an HSFC Joint National and Alberta Improving Hearth Health for Women New Investigator award (HSFC NNIA Davenport). B. Matenchuk is supported by a CIHR Doctoral Research Award and WCHRI Doctoral research Award. Á. Brislane is supported by a WCHRI Postdoctoral Research Award. These analyses stem from the larger PLASMA initiative (C.D. Steinback, M.H. Davenport).

## DISCLOSURES

None.

## Supporting information


**Appendix S1:** Supporting Information
