# Supplementary material for: Muscle sympathetic nerve activity during pregnancy: A systematic review and meta‐analysis
Source: Physiol Rep. 2023 Mar 10;11(5):e15626. doi: 10.14814/phy2.15626 (PMC10006587; doi:10.14814/phy2.15626)
Supplement: Supplementary file 1 — Appendix S1: Supporting Information [file PHY2-11-e15626-s001.doc]

**ONLINE SUPPLEMENT**

**Title: Muscle Sympathetic Nerve Activity during Pregnancy: A Systematic Review and Meta-Analysis**

**Authors:** Kelly M. GREENWALL^1^, Áine BRISLANE^1^, Brittany A. MATENCHUK^1^, Allison SIVAK^1^, and Margie H. DAVENPORT^1-^, Craig D. STEINBACK^1*^

**Supplementary Table 1:** Study Characteristics.

| ***Reference*** | ***N*** | ***Groupings*** | ***Country*** | ***Age*** | ***Gestational Age*** | ***Medications*** | ***Pre-Pregnancy BMI*** | ***Protocol*** |
| --- | --- | --- | --- | --- | --- | --- | --- | --- |
| Badrov 2019  Data linked to Fu 2017, Jarvis 2015, Stickford 2015 (baseline only) | 44 | 23 NP; 13 HRP; 8 subsequent GH | USA | NP: 30 ± 4.32  HRP: 31 ± 4.33  PIH: 29 ± 4.24 | Measured pre-pregnancy (midluteal phase),  early pregnancy (4-8 weeks),  late pregnancy (32-36 weeks), and postpartum (6-10 weeks post-delivery). | N/A | N/A | Baseline data obtained in resting supine  15° left lateral position during each testing period. |
| Charkoudian 2017  Also linked to Schmidt 2017, Usselman 2015 a, b | 46 | 23 NP; 23 NN | Canada | NP: 31 ± 3  NN: 28 ± 5 | 32 ± 4 | 8 NN were using oral contraceptives, 5 were using intrauterine devices, and one was using  Nuvaring. Two did not provide information. | 23 ± 3 | Baseline data collected with subjects in a semirecumbent position. |
| Fischer 2004 | 22 | 22 NP | Germany | 31.7 ± 3.9 | 22 ± 4 and 33 ± 5 weeks of gestation; 26 ± 6 weeks post-delivery. | None | N/A | Baseline data obtained in a 30° left lateral position during each testing period. |
| Folmar Sander 2003 | 47 | 20 NP; 17 PE; 10 NN  NB: for CPT, n = 11 for PIH, and n = 16 for NP. For postpartum: n = 16 for PIH, and n = 11 for NP. | Denmark | NP: 28.7 ± 4.02  PIH: 26.3 ± 6.18  NN: 28.7 ± 4.43 | NP: 34.5 ± 0.5  PIH: 35.8 ± 0.5  Both tested again at 3-6 months post-delivery | None | N/A | Baseline data collected in the left lateral supine position for 15 mins after a resting period of 45 mins. 2-min CPT. |
| Fu 2008a | 18 | 6 NP; 12 NN | USA | N/A | 4 – 7 weeks | N/A | N/A | Baseline data collected in supine position. Graded upright tilt of 30° and 60°. |
| Fu 2008b | 6 | 6 NP | USA | 24-35 | Measured pre-pregnancy, early pregnancy (<8 weeks), late pregnancy (not specified), and within 10 weeks post-delivery. | N/A | N/A | Baseline data collected in supine position. |
| Greenwood 1998  Data included in Greenwood 2003 but novel data on burst frequency. | 24 | 13 GH; 11 NN | England | PIH: 29.9 ± 5.77  NN: 27.7 ± 4.46 | PIH: 34.4 ± 1.6  NN: 34.1 ± 0.7 | 9 of 13 PIH women had commenced oral labetalol 12-48 h prior to study. | N/A | Baseline with subjects studied semi-supine  and rotated 30° into the left lateral position. CPT at 4°C  for ≥ 1 min; right hand IHG 30–40% of the MVC for 2 min. |
| Greenwood 2001  Complicated vs. uncomplicated pregnancy data included in Greenwood 2003 | 60 | 21 NN; 21 NP; 18 GH | England | NN: 28 ± 5.04  NP: 29 ± 3.67  PIH: 30 ± 5.52 | NP: 35 ± 0.6  PIH: 35 ± 0.9  Both tested again at 6 weeks postpartum | 9 of 18 patients with PIH had started oral labetalol 12-48 h prior. | N/A | Baseline with subjects studied semi-supine  and rotated 30° into the left lateral position. |
| Greenwood 2003  Previously published data in Greenwood 1998 and 2001 | 33 | 11 GH; 11 PE; 11 NP | England | PIH: 28 ± 4.97  PE: ± 27 4.97  NP: 28 ± 3.98 | PIH: 37 ± 0.5  PE: 35 ± 1.1  NP: 35 ± 0.6 | 5 and 6 of the women with PE and PIH, respectively, had started oral labetalol 12-48 h prior to study. | N/A | Baseline with subjects studied semi-supine and rotated 30° into the left lateral position. |
| Hissen 2017 | 1 | 1 NP | Australia | 28 | Measured pre-pregnancy, during 6-, 11-, 17-, 22-,  25-, 33- and 36-weeks of gestation, and 9 and 16 weeks post-delivery. | N/A | N/A | Baseline data collected with participant in a semi-recumbent  position with the left leg supported in an extension during each testing period. |
| Hissen 2020 | 35 | 18 Normal-weight Pregnant (NWP); 17 Obese Pregnant (OP) | USA | NWP: 30 ± 7  OP: 30 ± 5 | Measured during early pregnancy (8.8 ± 1.2 and 9.2 ± 0.9 for NWP and OP, respectively), and late pregnancy (32-34 weeks of gestation for both). | N/A | N/A | Baseline data collected supine for 5 min. |
| Hissen 2022 | 28 | 14 Normal-weight Pregnant (NWP); 14 Obese Pregnant (OP) | USA | NWP: 30 ± 6  OP: 28 ± 3 | Measured early pregnancy (5-12 weeks), late pregnancy (32-34 weeks), and post-pregnancy (6-10 weeks). | N/A | N/A | Baseline data collected in supine rest for 5 min. |
| Hsieh 2020 | 25 | 13 Normal-weight Pregnant (NWP); 12 Obese Pregnant (OP) | USA | N/A | Both ≤ 10 weeks | N/A | N/A | Baseline data collected in supine during rest in lab; sleep testing using WatchPat and 24-hr ambulatory BP performed at home. |
| Jarvis 2011 | 20 | 15 Caucasian pregnant; 5 African American pregnant | USA | N/A | Both 4-8 weeks | N/A | N/A | Baseline, 30° and 60° head‐up tilt (HUT), and recovery data collected. |
| Jarvis 2012 | 11 | 11 NP | USA | 29 ± 3 | Measured at pre-pregnancy (mid-luteal phase) and early pregnancy (6.2 ± 1.2 weeks of gestation). | None | 24.1 ± 4.6 | Baseline collected supine for 6 min. 30° and 60° head‐up tilt (HUT) for 5 min each or until presyncope, followed by a 3-min supine recovery during each testing period. |
| Merrill 1995 | 12 | 6 NP; 6 NN | USA | N/A | Both 3rd Trimester | N/A | N/A | Participants underwent progressive lower body negative pressure preceding baseline. |
| Okada 2015 | 21 | 12 Caucasian pregnant; 9 Asian pregnant | USA | Caucasian: 29 ± 3.46  Asian: 32 ± 3 | Measured at pre-pregnancy, early pregnancy (≤8 weeks), late pregnancy (32–36 weeks), and postpartum (6–10 weeks post-delivery). | N/A | Caucasian: 26.2 ± 7.62  Asian: 20.6 ± 3 | Baseline data collected in supine position for 6 min. 30° and 60° head‐up tilt (HUT) for 5 min each, followed by a 3 min supine recovery during each testing period. |
| Reyes 2018 | 2 | 2 NP | Canada | Participant 1: 36  Participant 2: 24 | Measured at pre-pregnancy (EF phase); 12-, 23-, -28, -38, weeks of gestation, and 2 months postpartum. | N/A | Participant 1: 22  Participant 2: 21 | Baseline data recorded for 10 min and 3-min CPT during each testing period. |
| Reyes 2020a  Partial overlap with Reyes 202b | 57 | 38 NP; 9 PE | Canada | NP: 32 ± 4  PE: 32 ± 5 | NP: 31 ± 4  PE: 31 ± 3 | 1 NP and 13 PE taking oral labetalol; 1 PE taking magnesium sulfate; 7 PE taking nifedipine; 4 NP and 9 PE taking insulin; 3 NP taking metformin; 1 NP and 3 PE taking levothyroxine; 4 NP and 2 PE taking progesterone; 3 PE taking Aspirin; 1 PE taking enoxaparin; and 1 PE taking oxybutynin. | NP: 25 ± 6  PE: 28 ± 5 | Baseline data obtained with participants in a 30° semi-recumbent position. 3-min CPT at ~0-4°C). 3-min hyperoxia in which participants breathed oxygen (FiO_2_ = 1.0; flow rate = 3L/min) through an oro-nasal mask. |
| Reyes 2020b  Partial overlap with Reyes 202a | 36 | 18 GDM; 18 NP | Canada | GDM: 33 ± 4  NP: 30 ± 4 | GDM: 33 ± 3  NP: 32 ± 5 | 6 GDM on insulin | GDM: 27.1 ± 5.7  NP: 25.1 ± 5.2 | Baseline, 3-minute CPT, 3-minute hyperoxia |
| Schmidt 2017  Data linked to Charkoudian 2017 | 37 | 18 NP; 19 NN | Canada | NP: 31 ± 3  NN: 27 ± 5 | NP: 33 ± 4 | 5 NN were taking hormonal contraceptives | NP: 23.9 ± 3.1  NN: 23.7 ± 4.9 | Baseline data collected with participants seated in a  semirecumbent position. 3-min CPT. NB: 13 NN and 10 NP underwent CPT. |
| Schobel 1996 | 30 | 9 PE; 8 NP; 6 NN; 7 HN | Germany | N/A | PE: 33 ± 1  NP: 32 ± 1  Postpartum recorded in 6 of 9 PE patients 1-3 months post-delivery. | 5 of the 9 patients with PE received  dihydralazine (Nepresol, Ciba; 25 mg taken orally). | PE: 26 ± 3  NP: 26 ± 2.83  NN: 25 ± 2.45 | Baseline data collected with subjects lying in a 30° left lateral position. 2-min CPT. Valsalva maneuver consisted of an expiratory pressure of 40 mm Hg for 15 seconds. |
| Skow 2020a | 33 | 19 NP; 14 NN | Canada | NP: 33 ± 3  NN: 32 ± 4 | 32 ± 3 | None | NP: 24.1 ± 4.8  NN: 25.2 ± 2.7 | Baseline data obtained with subjects seated semi-reclined at 45º. IHG performed 30% of MVC for 2 mins. 2-min post-exercise circulatory occlusion  (PECO). |
| Skow 2020b | 59 | 31 exercise pregnant; 28 control pregnant | Canada | Exercise: 31 ± 2  Control: 32 ± 4 | Exercise: 18 ± 2 (pre-intervention) and 34 ± 1 (post-intervention)  Control: 18 ± 1 (pre-intervention) and 35 ± 2 (post-intervention) | N/A | Exercise: 26.8 ± 7.8  Control: 24.6 ± 4.8 | Baseline data collected in semi-reclined position during each testing period. 3-min CPT at 4ºC. |
| Stickford 2015a  Baseline linked to Badrov 2019 | 16 | 8 history of PE; 8 NP | USA | N/A | 4 – 8 weeks | N/A | N/A | Baseline data collected during 6 mins of supine rest, followed by 6 mins of 60° of head-up tilt. |
| Stickford 2015b  Baseline linked to Badrov 2019 | 41 | 33 pre-pregnancy (13 OW/OB_  41 early (13 OW/OB)  31 late (9 OW/OB) | USA | N/A | Measured pre-pregnancy (mid-luteal), early pregnancy (4-8 weeks), late pregnancy (32-36 weeks), and post-pregnancy (6-10 weeks). | N/A | N/A | Baseline data collected for 6 mins of rest after >30 mins of supine position. |
| Yoo 2020 | 43 | 21 NP; 22 NN | USA | N/A | 6 - 10 weeks | N/A | N/A | Participants underwent static handgrip exercise to fatigue at 40% MVC force proceeded by 2 mins of post-exercise circulatory arrest. |

NP = Normotensive Pregnancy

NN = Normotensive Nonpregnant

PIH = Pregnancy-induced Hypertension

HRP = High-risk Normotensive Pregnant

HN = Hypertensive Nonpregnant

GDM = Gestational Diabetes Mellitus

GH = Gestational Hypertension

PE = Preeclampsia

PP = Postpartum

**Duplicate Papers:**

Reyes L, Badrov M, Fu Q, Steinback CD, Davenport MH. Influence of age, body mass index and weight gain on sympathetic activity during pregnancy. *Applied Physiology, Nutrition and Metabolism* 45(9):1041-1044.

Badrov M, Yoo JK, Steinback CD, Davenport MH, Fu Q. Influence of multi-parity on sympathetic nerve activity during normal pregnancy. *AJP Heart and Circulatory Physiology* 318(4):H816-H819, 2020.

Fu Q, Park S, Yoo JK, Hieda M, Okada Y, Jarvis SS, Stickford AS, Best SA, Levine BA. Role of corin in blood pressure regulation during pregnancy in humans. *Clin Auton Res* 27: 295-353, 2017.

Steinback CD, Fraser G, Usselman CW, Reyes LM, Julian C, Stickland MK, Chari R, Khurana R, Davidge S, Davenport MH. Blunted Sympathetic neurovascular transduction during normotensive pregnancy. *J Physiol* 597(14):3687-3696, 2019.

Stickford ASL, Parker RS, Best SA, Okada Y, Jarvis SS, Roberts MA, Fu Q. Muscle sympathetic nerve activity is related to body mass during late pregnancy. *Med Scie Sports Exercise* 47(5S): 153-154, 2015.

Usselman CW, Wakefield PK, Skow RJ, Stickland MK, Chari RS, Julian CG, Steinback CD*, Davenport MH*. Regulation of sympathetic nerve activity during the cold pressor test in normotensive pregnant and nonpregnant women. *Hypertension*, *66*(4):858-64, 2015.

Usselman CW*,* Skow RJ*,* Matenchuk BA, Chari RS, Julian CG, Stickland MK, Davenport MH*, Steinback CD*. Sympathetic baroreflex gain in normotensive pregnant women. *J Appl Physiol,* *119*(5):468-74, 2015.

**Steps taken to prevent over-counting:**

Reyes et al 2020a & b:

All individuals in the GDM paper control group were also in the PE paper. To prevent double counting of participants, the total number of participants that were the same between both studies were removed from the total (n=9) in the uncomplicated group and divided by two. Half of the number was added back to the control group in each study (n=25 PE 2020; n=14 GDM 2020 for SNA); (n= 34 PE 2020; n= 14 GDM 2020 for cardiovascular data).

Greenwood 1998/2001/2003:

All individuals in the 2003 normal pregnancy paper were also in 2001. To prevent double counting of participants, the total number of participants that were the same between both studies were removed from the total (n=11) in the uncomplicated group and divided by two. Half of the number was added back to the control group in each study (n=6 2003; n= 16 2001)

|  | 1998 - duplicate of 2001 BUT they have CPT and IHG change scores | 2001 – no reactivity | 2003 – no reactivity |
| --- | --- | --- | --- |
| BF | **10 normal preg, 12 PIH** | X | x |
| BI | 10 normal preg, 12 PIH | **21non-p 21preg 18 PIH** | 11 preg, 11 PIH, **11 PE** |
| MAP | 10 normal preg, 12 PIH | **21non-p 21preg 18 PIH** | 11 preg, 11 PIH, **11 PE** |
| SBP | 10 normal preg, 12 PIH | **21non-p 21preg 18 PIH** | 11 preg, 11 PIH, **11 PE** |
| DBP | 10 normal preg, 12 PIH | **21non-p 21preg 18 PIH** | 11 preg, 11 PIH, **11 PE** |
| HR | 10 normal preg, 12 PIH | **21non-p 21preg 18 PIH** | 11 preg, 11 PIH, **11 PE** |

**Authors contacted for additional information:**

The authors would like to thank Dr. Qi Fu for generously providing information about duplicate publications, as well as additional data.

Dr. John Greenwood and Dr. Kirsten Foltmar-Sander were contacted; Dr. Greenwood was unable to provide additional data, we received no response from Dr. Foltmar-Sander.

**Supplementary Table 2:** Joanna Briggs Institute Critical Appraisal of Evidence Effectiveness Cross Sectional data.

| FULL TEXT | **Were the criteria for inclusion in the sample clearly defined?** | **Were the study subjects and the setting described in detail?** | **Was the exposure measured in a valid and reliable way?** | **Were objective, standard criteria used for measurement of the condition?** | **Were confounding factors identified?** | **Were strategies to deal with confounding factors stated?** | **Were the outcomes measured in a valid and reliable way?** | **Was appropriate statistical analysis used?** |
| --- | --- | --- | --- | --- | --- | --- | --- | --- |
| FU 2008 | yes | no | yes | yes | no | no | yes | unclear |
| GREENWOOD 1998 | yes | yes | yes | yes | yes | yes | yes | yes |
| GREENWOOD 2003 | yes | yes | yes | yes | yes | yes | yes | yes |
| SCHMIDT 2018 | yes | yes | yes | yes | yes | yes | yes | yes |
| GREENWOOD 2001 | yes | yes | yes | yes | yes | yes | yes | yes |
| CHARKOUDIAN 2017 | yes | yes | yes | yes | yes | yes | yes | yes |
| SCHOBEL 1996 | yes | yes | yes | yes | yes | yes | yes | yes |
| FU 2008 - ABSTRACT | yes | no | yes | yes | no | no | yes | unclear |
| JARVIS 2011 - ABSTRACT | yes | no | yes | yes | no | no | yes | unclear |
| FOLTMAR-SANDER 2003 | yes | yes | yes | yes | yes | yes | yes | yes |
| HISSEN 2020 - ABSTRACT/POSTER | yes | yes | yes | yes | yes | no | yes | unclear |
| 1442 | yes | yes | yes | yes | yes | yes | yes | yes |
| SKOW 2020 | yes | yes | yes | yes | yes | yes | yes | yes |
| REYES 2020 | yes | yes | yes | yes | yes | yes | yes | yes |
| 1476 - ABSTRACT | yes | no | yes | yes | yes | yes | yes | unclear |
| 1477 | yes | yes | yes | yes | no | no | yes | unclear |
| 1478 | yes | no | yes | yes | yes | yes | yes | unclear |
|
|

**Supplementary Table 3:** Joanna Briggs Institute Critical Appraisal of Evidence Effectiveness Case Report data.

| FULL TEXT | **Were patient's demographic characteristics clearly described?** | **Was the patient's history clearly described and presented as a timeline?** | **Was the current clinical condition of the patient on presentation clearly described?** | **Were diagnostic tests or assessment methods and the results clearly described?** | **Was the intervention(s) or treatment procedure(s) clearly described?** | **Was the post-intervention clincial condition clearly described?** | **Were adverse events (harms) or unanticipated events identified and described?** | **Does the case report provide takeaway lessons?** |
| --- | --- | --- | --- | --- | --- | --- | --- | --- |
| REYES 2018 | yes | yes | yes | yes | yes | yes | no | yes |
| HISSEN 2017 | yes | yes | yes | yes | yes | yes | no | yes |

**Supplementary Table 4:** Joanna Briggs Institute Critical Appraisal of Evidence Effectiveness Cohort

| FULL TEXT | **Were the two groups similar and recruited from the same population?** | **Were the exposures measured similarily to assign people to both exposed and unexposed groups?** | **Was the exposure measured in a valid and reliable way?** | **Were confounding factors identified?** | **Were strategies to deal with confounding factors stated?** | **Were the groups/participants free of the outcome at the start of the study (or at the moment of exposure)?** | **Were the outcomes measured in a valid and reliable way?** | **Was the follow up time reported and sufficient to be long enough for outcomes to occur?** | **Was follow up complete, and if not, were the reasons to loss to follow up described and explored?** | **Were strategies to address incomplete follow up utilized?** | **Was appropriate statistical analysis used?** |
| --- | --- | --- | --- | --- | --- | --- | --- | --- | --- | --- | --- |
| HISSEN 2022 abstract | Yes | yes | yes | yes | no | yes | yes | yes | no | no | Unclear (not stated) |
| BADROV 2019 | yes | yes | yes | yes | yes | yes | yes | yes | no | no | yes |
| FISCHER 2004 | yes | yes | yes | yes | no | yes | yes | yes | yes | yes | yes |
| OKADA 2014 | yes | yes | yes | yes | yes | yes | yes | yes | yes | yes | yes |
| JARVIS 2012 | yes | yes | yes | yes | yes | yes | yes | yes | yes | yes | yes |
| Abstract | yes | yes | yes | yes | yes | yes | yes | yes | no | no | unclear (not stated) |
| Stickford acsm 2015 | yes | yes | yes | yes | yes | yes | yes | yes | no | no | Yes |

**Supplementary Table 5:** Joanna Briggs Institute Critical Appraisal of Evidence Effectiveness RCT

| FULL TEXT | **Was true randomization used for assignment of participants to treatment groups?** | **Was allocation to treatment groups concealed?** | **Were treatment groups similar at the baseline?** | **Were participants blind to treatment asignment?** | **Were those delivering treatment blind to treatment assignment?** | **Were outcomes assessors blind to treatment assignment?** | **Were treatment groups treated identically other than the intervention of interest?** | **Was follow up complete and if not, were differences between groups in terms of their follow up adequately described and analyzed?** | **Were participants analyzed in the groups to which they were randomized?** | **Were outcomes measured in the same way for treatment groups?** | **Were outcomes measured in a reliable way?** | **Was appropriate statistical analysis used?** | **Was the trial design appropriate, and any deviations from the standard RCT design (individual randomization, parallel groups) accounted for in the conduct and analysis of the trial?** |
| --- | --- | --- | --- | --- | --- | --- | --- | --- | --- | --- | --- | --- | --- |
| FU 2017 - ABSTRACT | yes | Yes | yes | no | no | yes | yes | yes | yes | yes | yes | yes | yes |


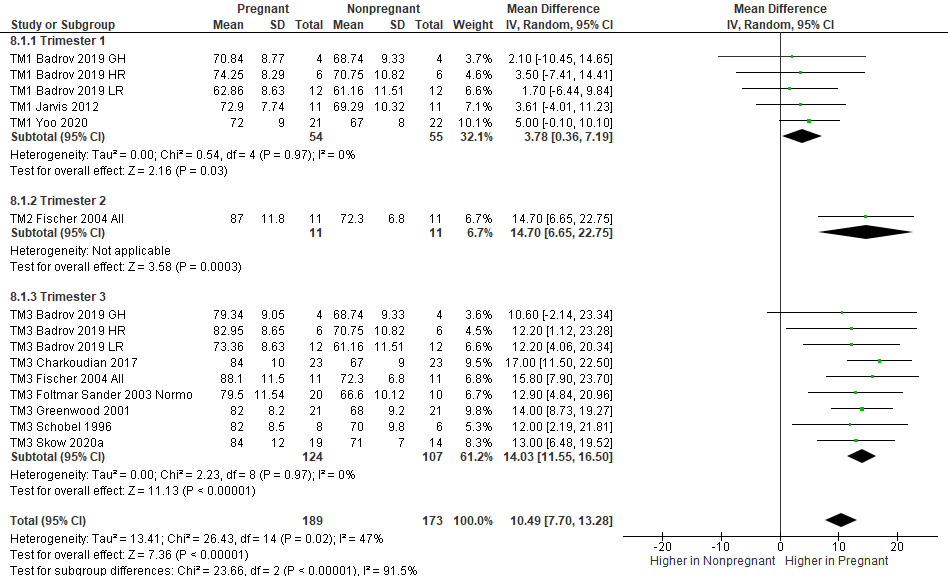


Online Supplement Figure 1: Effects of trimester of pregnancy compared to non-pregnant controls on heart rate. MD values are in units per minute. df, degrees of freedom; IV, inverse variance; HR, high risk; LR, low risk; GH, subsequently developed gestational hypertension; TM1, trimester 1; TM3, trimester 3.


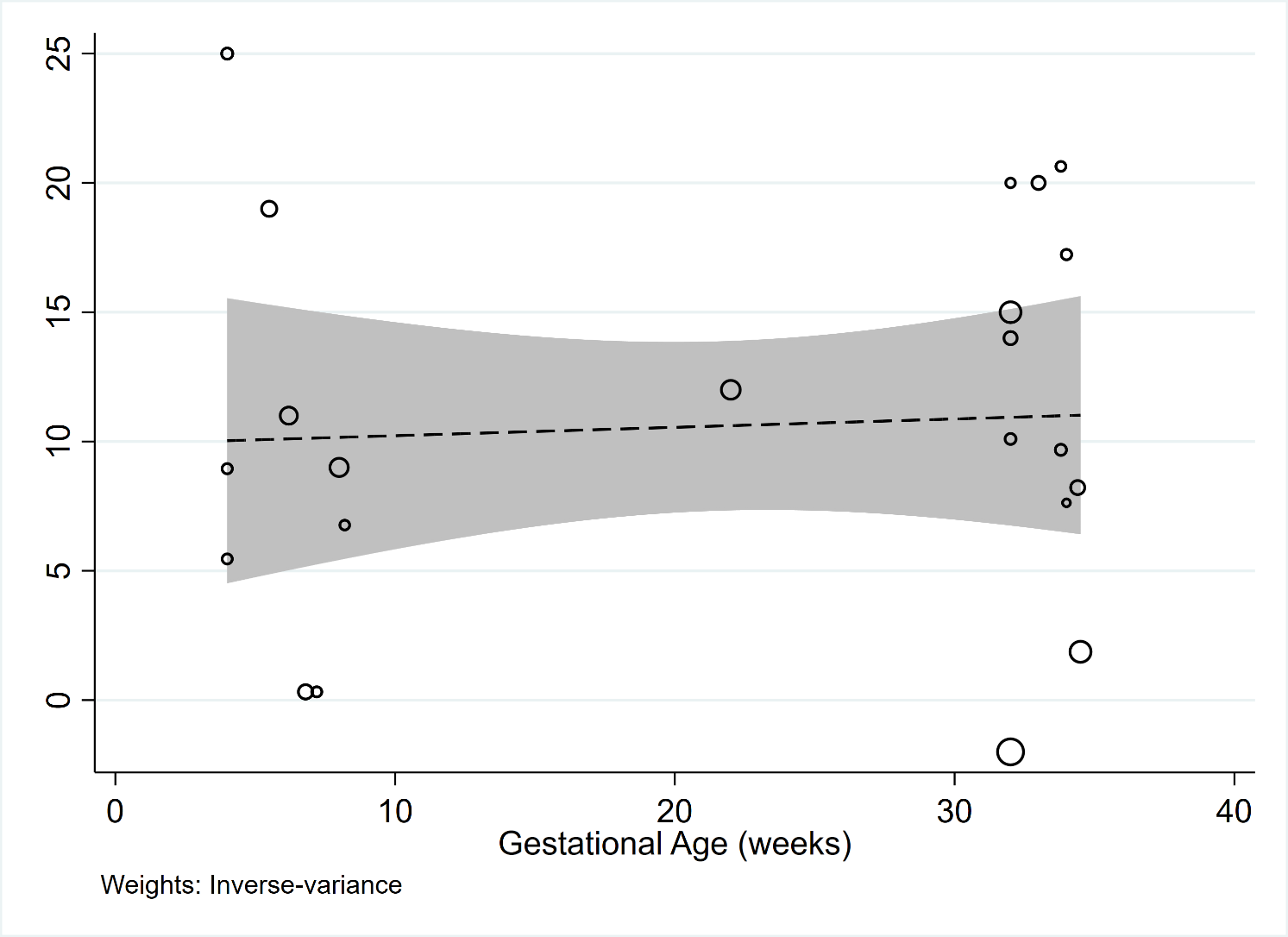


∆ Burst Frequency (bursts/min)

Online Supplement Figure 2: Relationship between MSNA burst frequency and gestational age (weeks) in uncomplicated pregnancy. No significant relationship between MSNA burst frequencyand gestational age was observed in uncomplicated pregnancy but not non-pregnant individuals. Weighted meta-regressions are denoted by the dashed line, with 95% confidence intervals indicated by the shaded region. The size of each dot represents the weight of the particular study within the regression analysis.


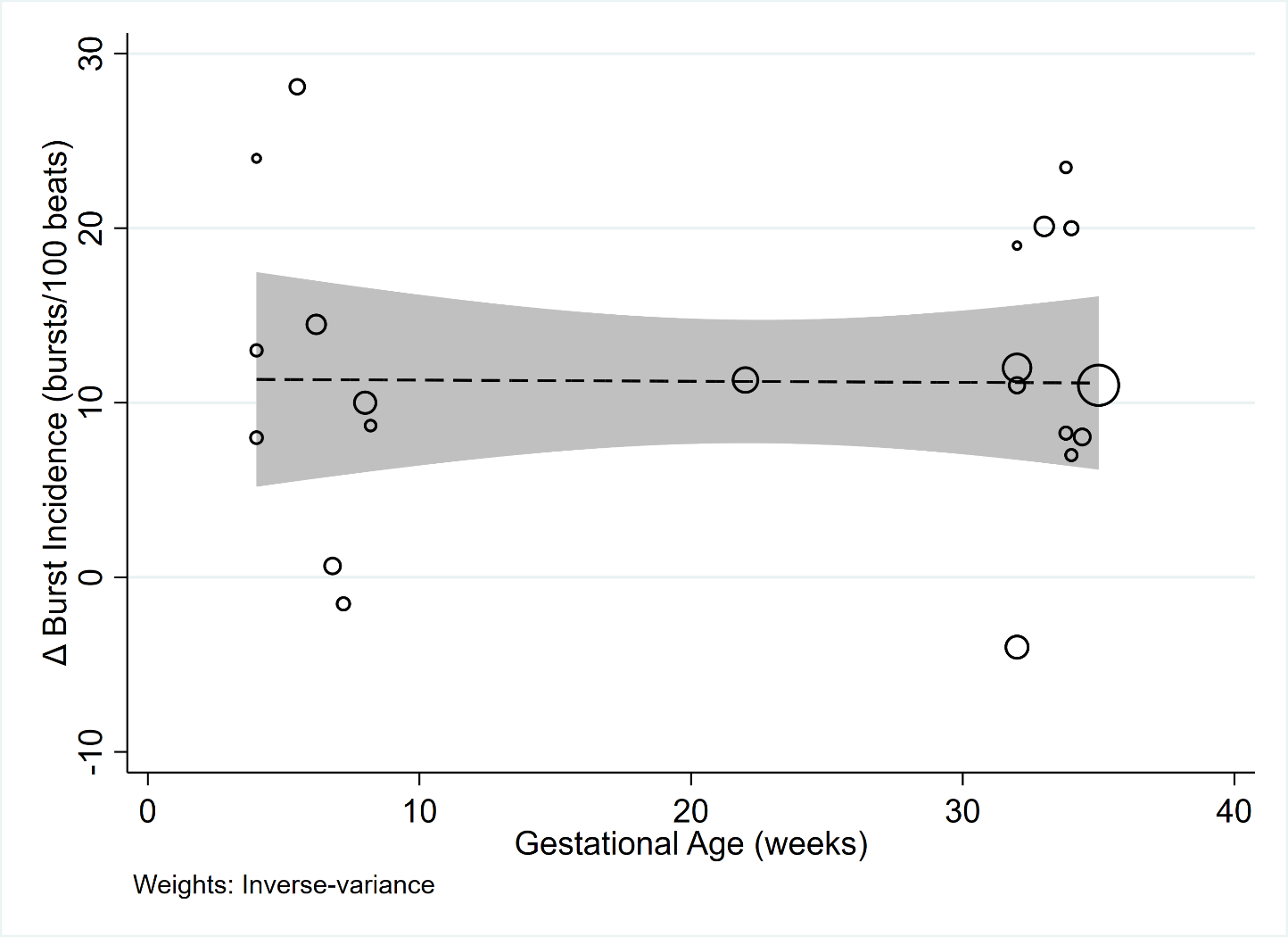


Online Supplement Figure 3: Relationship between MSNA burst incidence and gestational age (weeks) in uncomplicated pregnancy. No significant relationship between MSNA burst incidence and gestational age was observed in uncomplicated pregnancy but not non-pregnant individuals. Weighted meta-regressions are denoted by the dashed line, with 95% confidence intervals indicated by the shaded region. The size of each dot represents the weight of the particular study within the regression analysis.


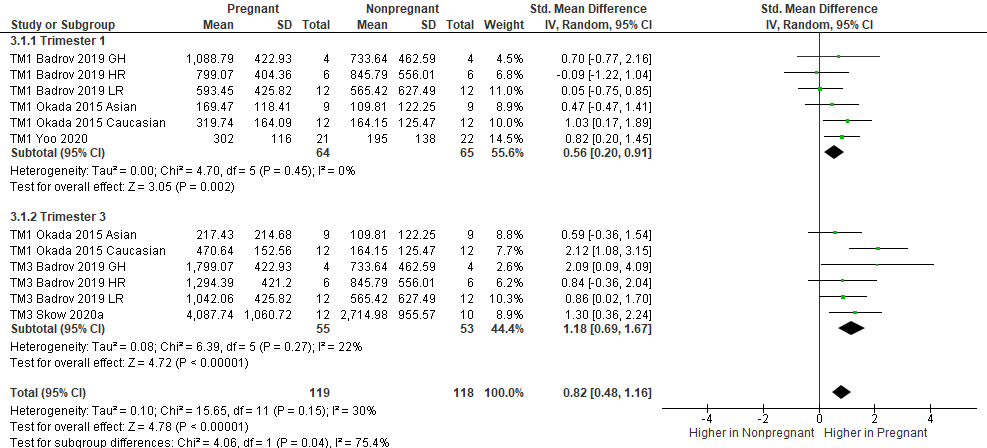


Online Supplement Figure 4: Effects of trimester of pregnancy compared to non-pregnant controls on total muscle sympathetic nerve activity. df, degrees of freedom; IV, inverse variance; HR, high risk; LR, low risk; GH, subsequently developed gestational hypertension; TM1, trimester 1; TM3, trimester 3.


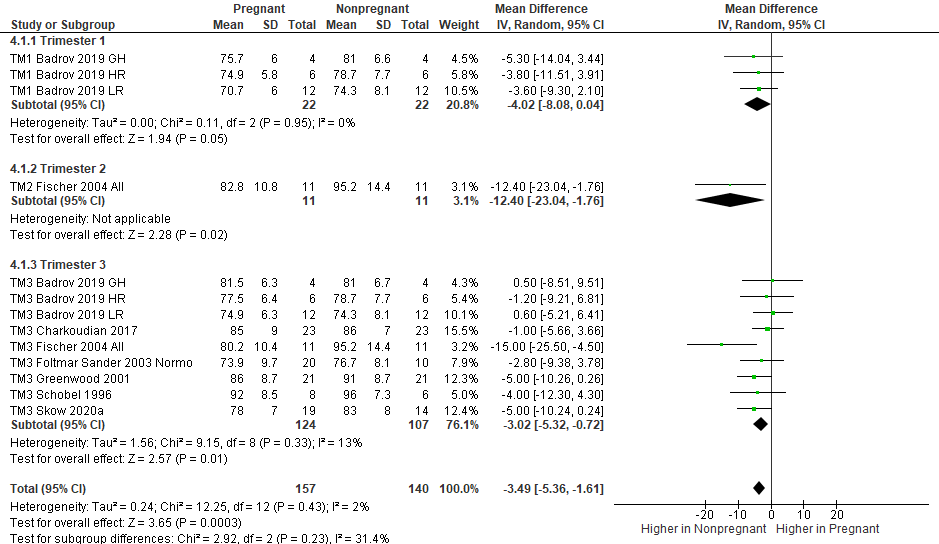


Online Supplement Figure 5: Effects of trimester of pregnancy compared to non-pregnant controls on mean arterial pressure. MD values are in units per minute. df, degrees of freedom; IV, inverse variance; HR, high risk; LR, low risk; GH, subsequently developed gestational hypertension; TM1, trimester 1; TM3, trimester 3.


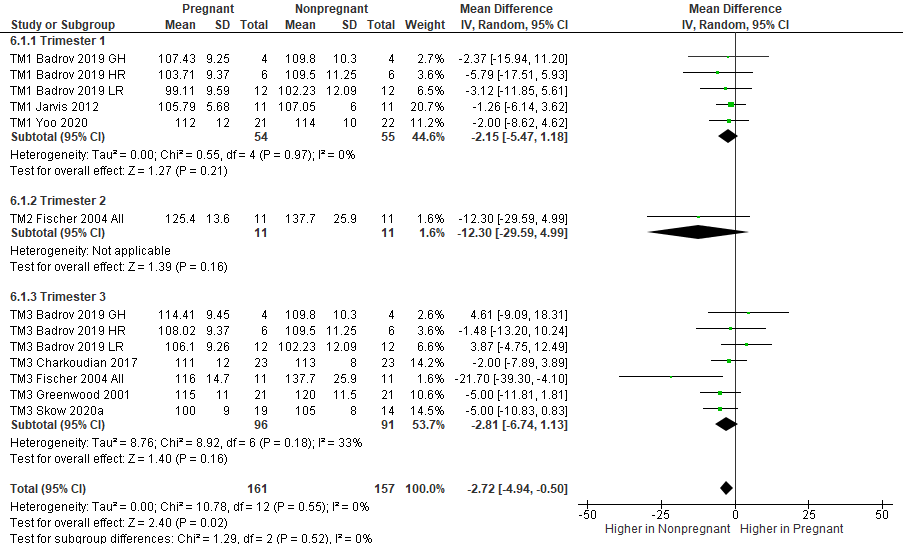


Online Supplement Figure 6: Effects of trimester of pregnancy compared to non-pregnant controls on systolic blood pressure. MD values are in units per minute. df, degrees of freedom; IV, inverse variance; HR, high risk; LR, low risk; GH, subsequently developed gestational hypertension; TM1, trimester 1; TM3, trimester 3.


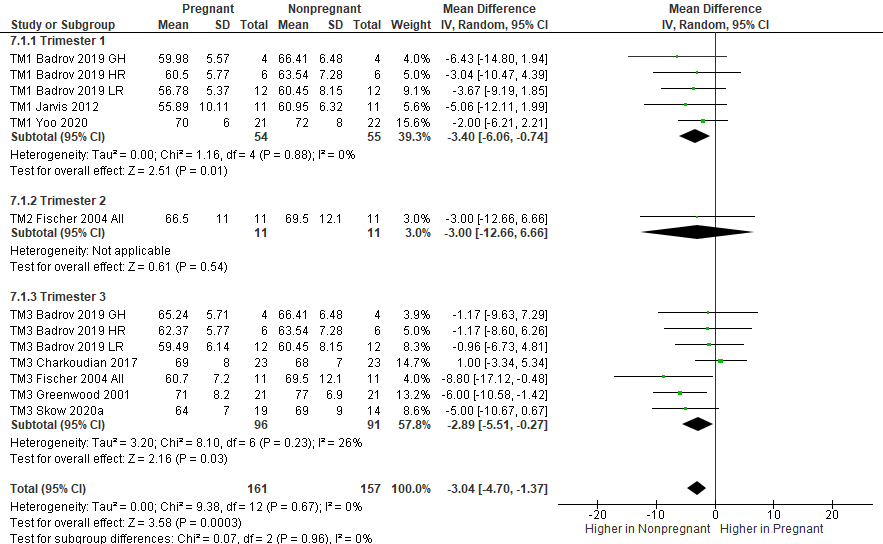


Online Supplement Figure 7: Effects of trimester of pregnancy compared to non-pregnant controls on diastolic blood pressure. MD values are in units per minute. df, degrees of freedom; IV, inverse variance; HR, high risk; LR, low risk; GH, subsequently developed gestational hypertension; TM1, trimester 1; TM3, trimester 3.


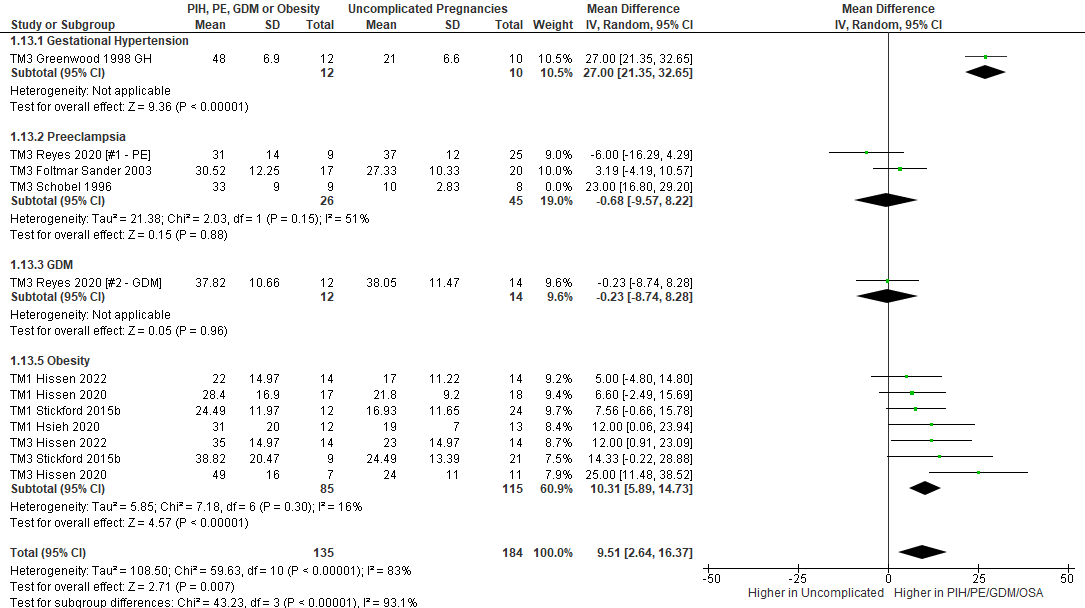


Online Supplement Figure 8: Basal MSNA burst frequency (bursts per minute) in uncomplicated and complicated pregnancies. Sensitivity analysis with Schobel 1996 removed from the analysis. df, degrees of freedom; IV, inverse variance; PE, preeclampsia; GDM, gestational diabetes melitus. NOTE: Greenwood 2001 utilzed old terminology (pregnancy induced hypertension) that falls within the current definition of gestational hypertension.


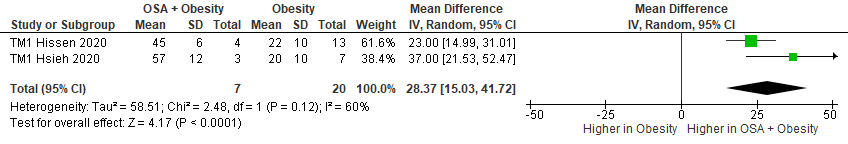


Online Supplement Figure 9: Basal MSNA burst frequency (bursts per minute) in individuals with Obstructive Sleep Apnea + obesity compared to those with obesity. df, degrees of freedom; IV, inverse variance.


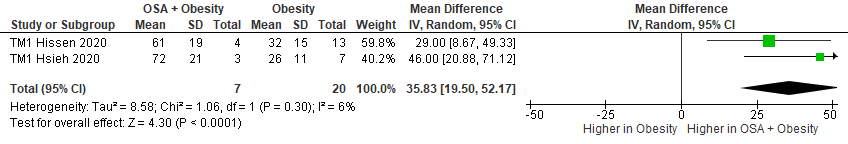


Online Supplement Figure 10: Basal MSNA burst incidence (bursts/100 heart beats) in individuals with Obstructive Sleep Apnea + obesity compared to those with obesity. df, degrees of freedom; IV, inverse variance.


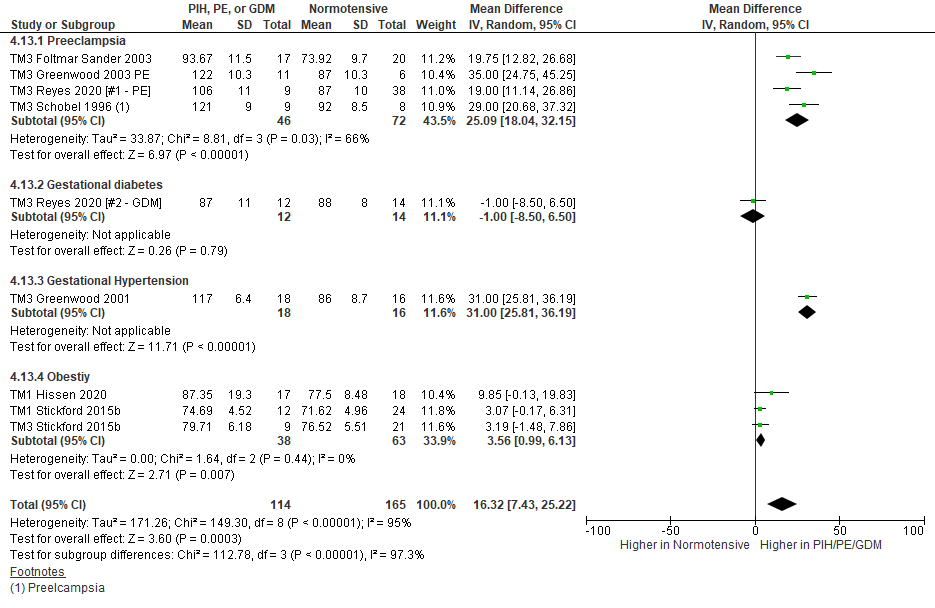


Online Supplement Figure 11: Basal mean arterial pressure in uncomplicated and complicated pregnancies. df, degrees of freedom; IV, inverse variance; PE, preeclampsia; NOTE: Greenwood 2001 utilzed old terminology (pregnancy induced hypertension) that falls within the current definition of gestational hypertension.


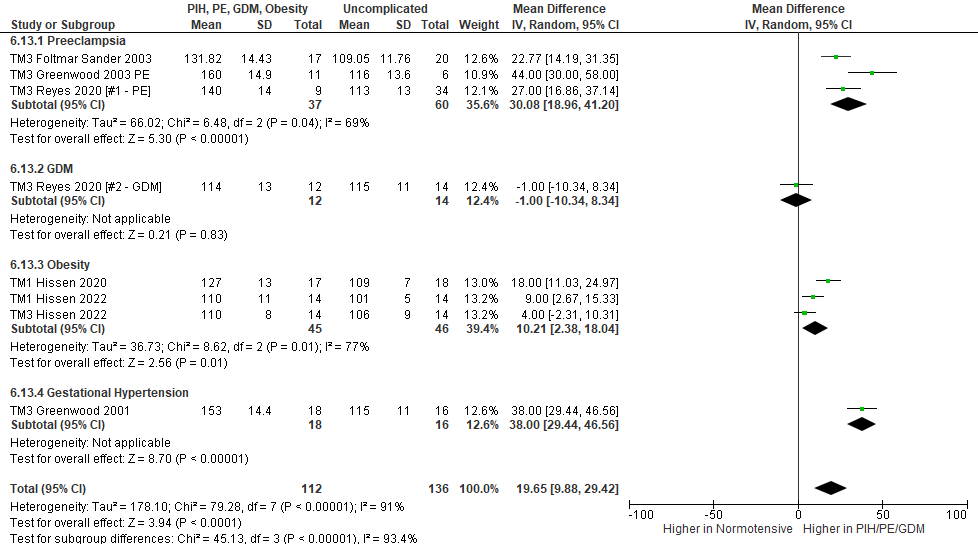


Online Supplement Figure 12: Basal systolic blood pressure in uncomplicated and complicated pregnancies. df, degrees of freedom; IV, inverse variance; PE, preeclampsia; NOTE: Greenwood 2001 utilzed old terminology (pregnancy induced hypertension) that falls within the current definition of gestational hypertension.


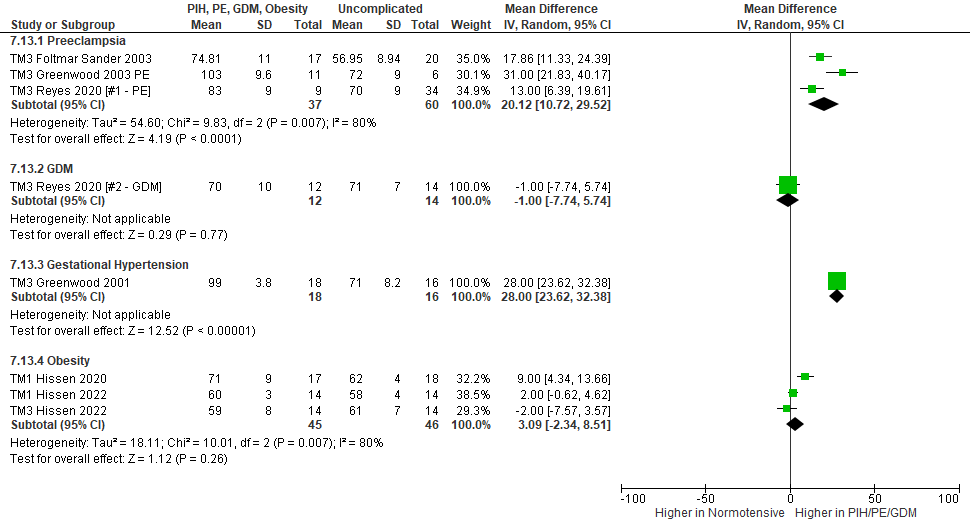


Online Supplement Figure 13: Basal diastolic blood pressure in uncomplicated and complicated pregnancies. df, degrees of freedom; IV, inverse variance; PE, preeclampsia; NOTE: Greenwood 2001 utilzed old terminology (pregnancy induced hypertension) that falls within the current definition of gestational hypertension.


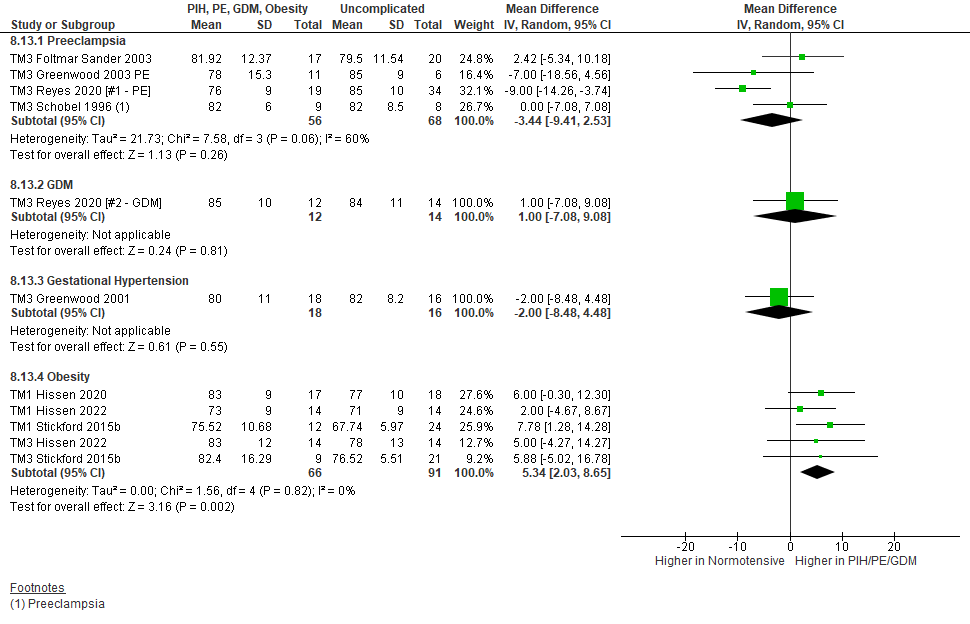


Online Supplement Figure 14: Basal heart rate in uncomplicated and complicated pregnancies. df, degrees of freedom; IV, inverse variance; PE, preeclampsia; NOTE: Greenwood 2001 utilzed old terminology (pregnancy induced hypertension) that falls within the current definition of gestational hypertension.


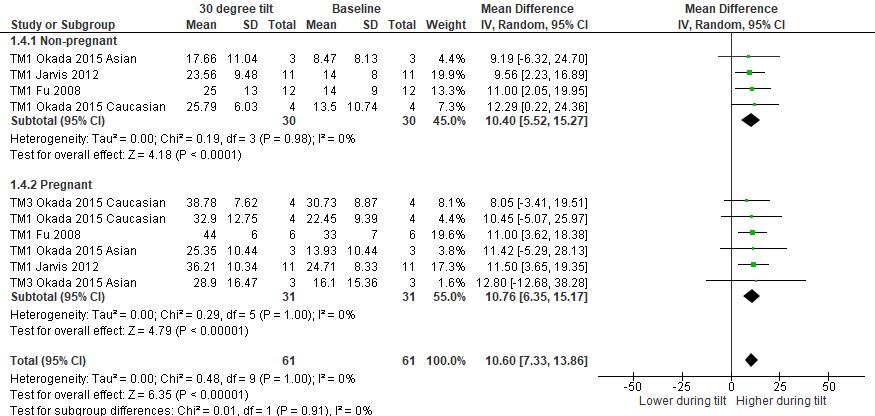


Online Supplement Figure 15: Change in burst frequency from supine to 30° upright tilt between pregnant and non-pregnant individuals. MD values are in units per minute. df, degrees of freedom; IV, inverse variance; TM1, trimester 1; TM3, trimester 3.


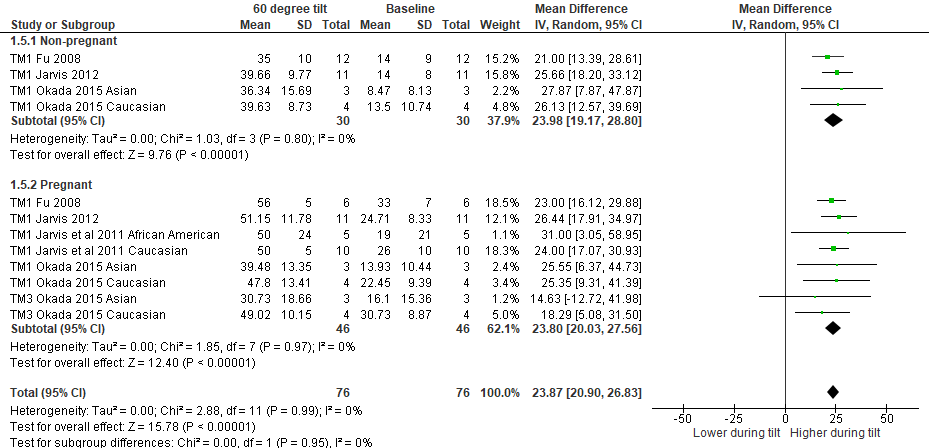


Online Supplement Figure 16: Change in burst frequency from supine to 60° upright tilt between pregnant and non-pregnant individuals. MD values are in units per minute. df, degrees of freedom; IV, inverse variance; TM1, trimester 1; TM3, trimester 3.


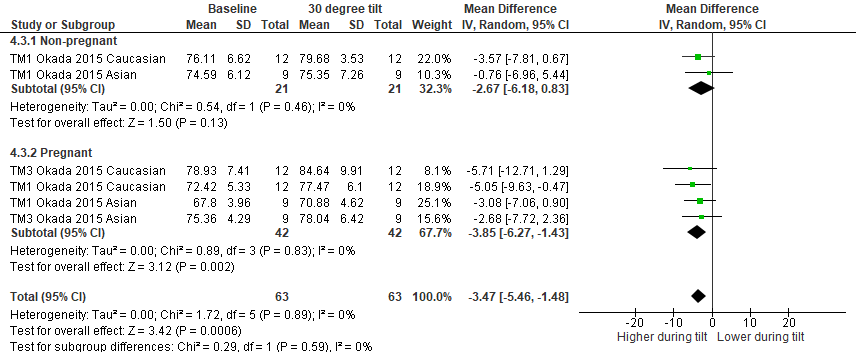


Online Supplement Figure 17: Change in mean arterial pressure from supine to 30° upright tilt between pregnant and non-pregnant individuals. MD values are in units per minute. df, degrees of freedom; IV, inverse variance; TM1, trimester 1; TM3, trimester 3.


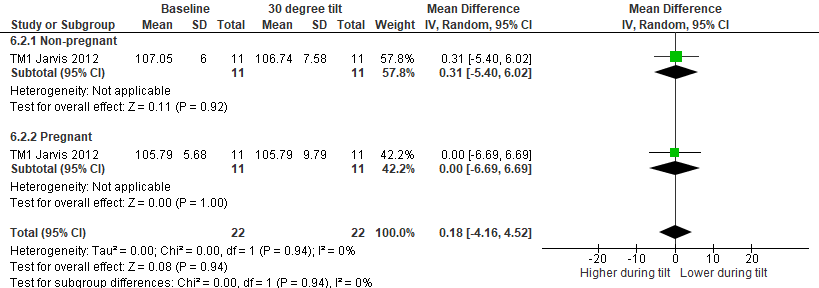


Online Supplement Figure 18: Change in systolic blood pressure from supine to 30° upright tilt between pregnant and non-pregnant individuals. MD values are in units per minute. df, degrees of freedom; IV, inverse variance; TM1, trimester 1; TM3, trimester 3.


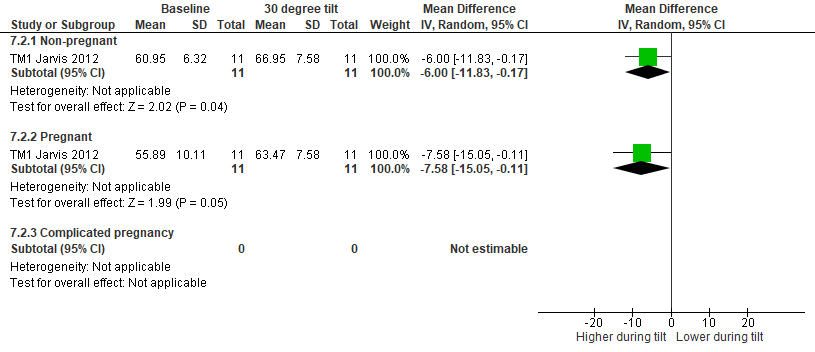


Online Supplement Figure 19: Change in diastolic blood pressure from supine to 30° upright tilt between pregnant and non-pregnant individuals. MD values are in units per minute. df, degrees of freedom; IV, inverse variance; TM1, trimester 1; TM3, trimester 3.


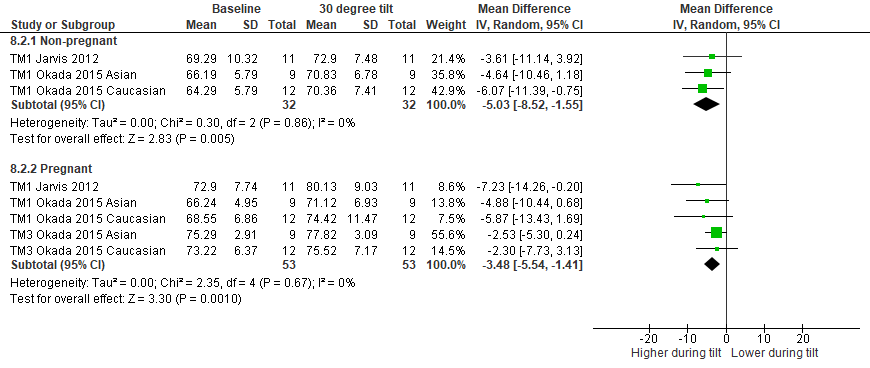


Online Supplement Figure 20: Change in heart rate from supine to 30° upright tilt between pregnant and non-pregnant individuals. MD values are in units per minute. df, degrees of freedom; IV, inverse variance; TM1, trimester 1; TM3, trimester 3.


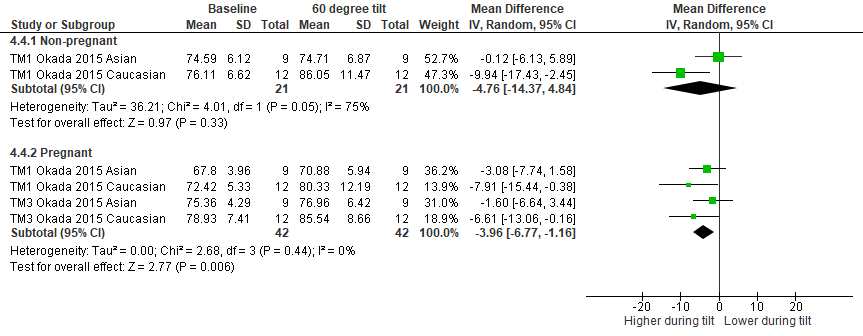


Online Supplement Figure 21: Change in mean arterial pressure from supine to 60° upright tilt between pregnant and non-pregnant individuals. MD values are in units per minute. df, degrees of freedom; IV, inverse variance; TM1, trimester 1; TM3, trimester 3.


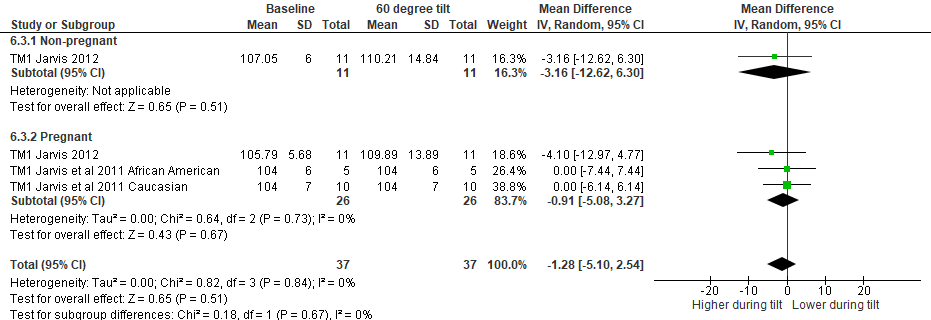


Online Supplement Figure 22: Change in systolic blood pressure from supine to 60° upright tilt between pregnant and non-pregnant individuals. MD values are in units per minute. df, degrees of freedom; IV, inverse variance; TM1, trimester 1; TM3, trimester 3.


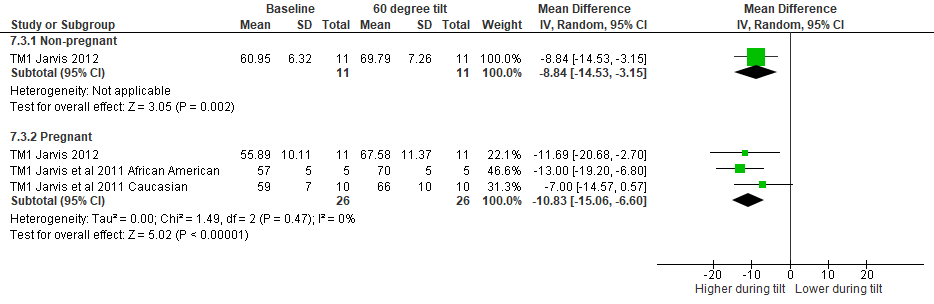


Online Supplement Figure 23: Change in diastolic blood pressure from supine to 60° upright tilt between pregnant and non-pregnant individuals. MD values are in units per minute. df, degrees of freedom; IV, inverse variance; TM1, trimester 1; TM3, trimester 3.


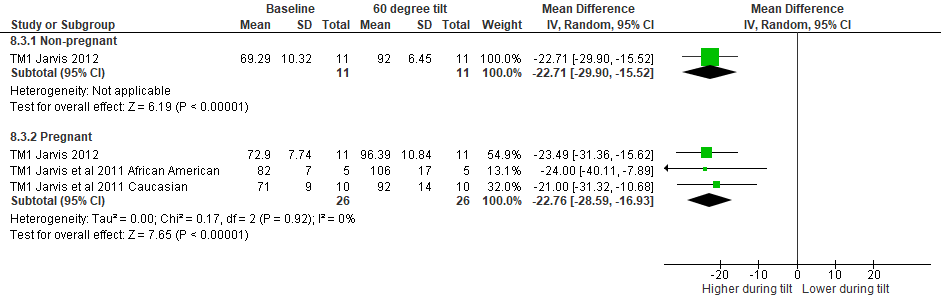


Online Supplement Figure 24: Change in heart rate from supine to 60° upright tilt between pregnant and non-pregnant individuals. MD values are in units per minute. df, degrees of freedom; IV, inverse variance; TM1, trimester 1; TM3, trimester 3.


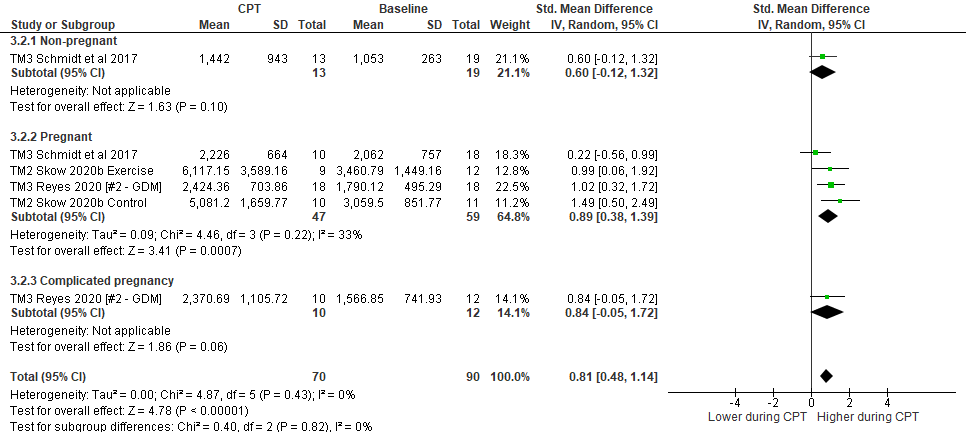


Online Supplement Figure 25 Change in total muscle sympathetic nerve activity in response to a cold pressor test. df, degrees of freedom; IV, inverse variance; TM1, trimester 1; TM2; TM3, trimester 3; GDM, gestational diabetes mellitus.


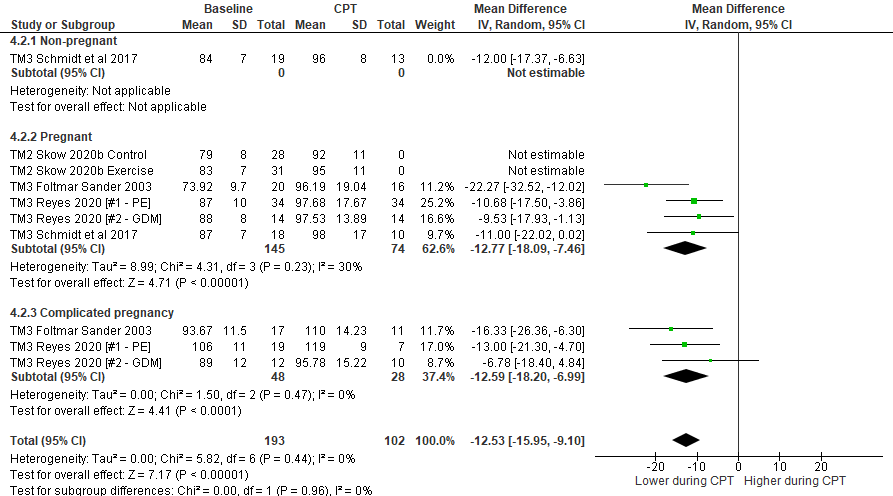


Online Supplement Figure 26: Change in mean arterial pressure in response to a cold pressor test. df, degrees of freedom; IV, inverse variance; TM1, trimester 1; TM2; TM3, trimester 3; GDM, gestational diabetes mellitus.


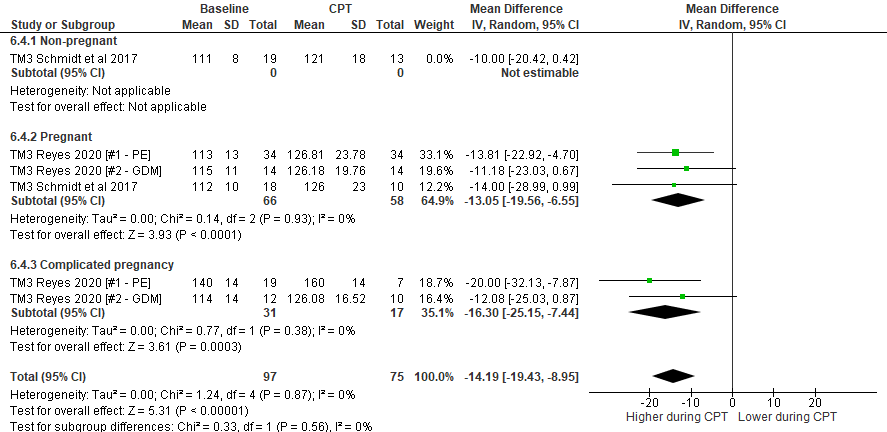


Online Supplement Figure 27: Change in systolic blood pressure in response to a cold pressor test. df, degrees of freedom; IV, inverse variance; TM1, trimester 1; TM2; TM3, trimester 3; GDM, gestational diabetes mellitus.


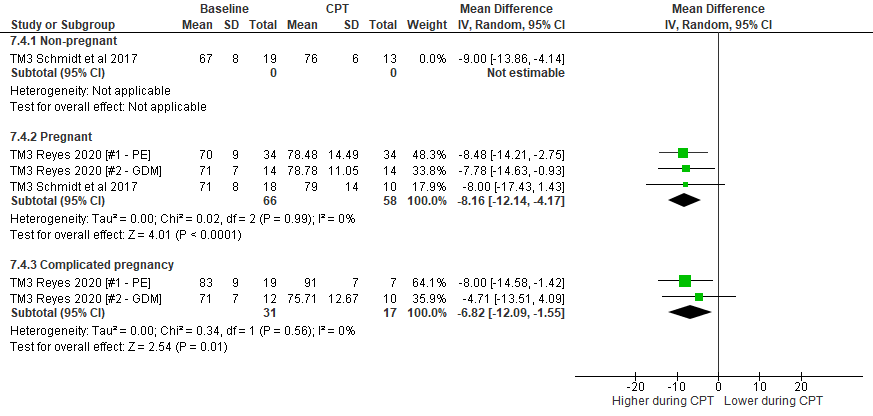


Online Supplement Figure 28: Change in systolic blood pressure in response to a cold pressor test. df, degrees of freedom; IV, inverse variance; TM1, trimester 1; TM2; TM3, trimester 3; GDM, gestational diabetes mellitus.


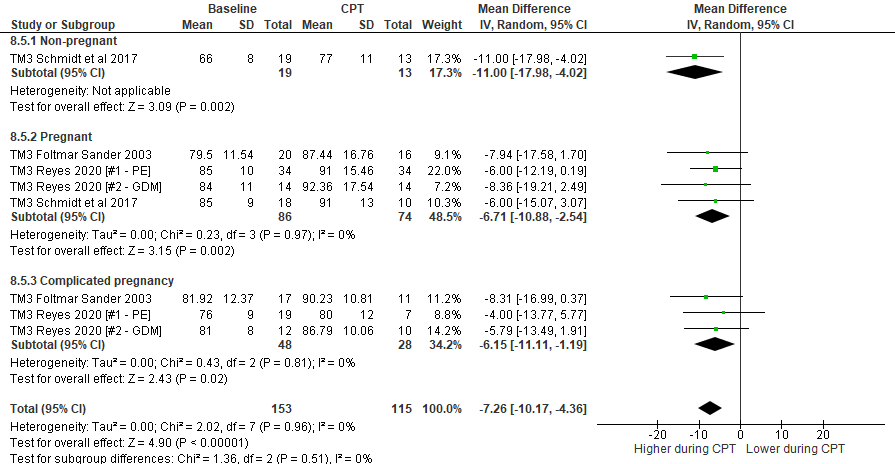


Online Supplement Figure 29: Change in heart rate in response to a cold pressor test. df, degrees of freedom; IV, inverse variance; TM1, trimester 1; TM2; TM3, trimester 3; GDM, gestational diabetes mellitus.


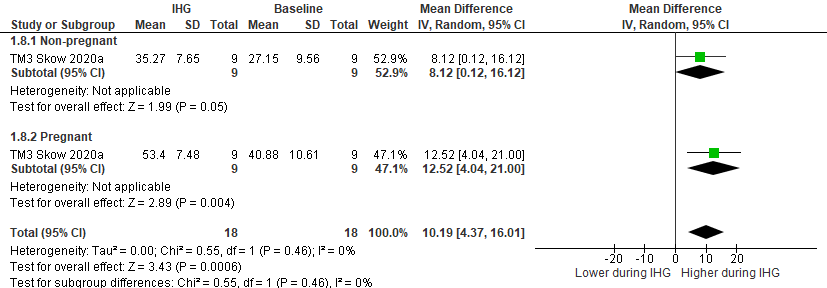


Online Supplement Figure 30: Change in burst frequency in response to an isometric handgrip between pregnant and non-pregnant women. df, degrees of freedom; IV, inverse variance; TM1, trimester 1; TM2; TM3, trimester 3.


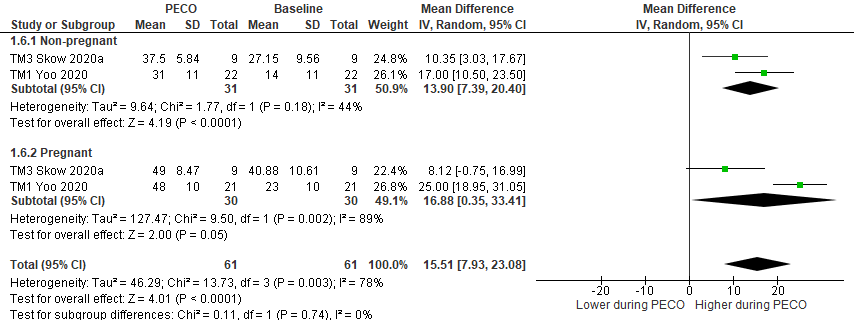


Online Supplement Figure 31: Change in burst frequency in response to a post-exercise circulatory occlusion between pregnant and non-pregnant women. df, degrees of freedom; IV, inverse variance; TM1, trimester 1; TM2; TM3, trimester 3.


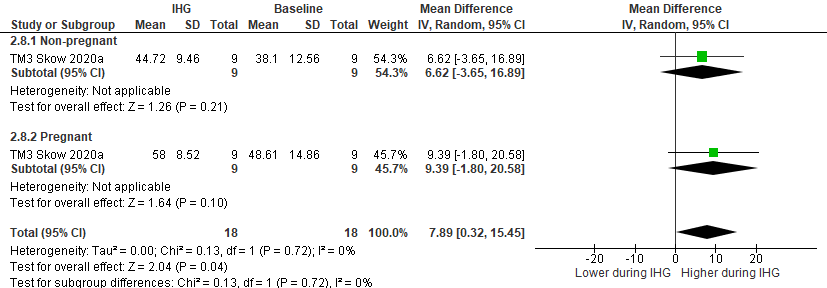


Online Supplement Figure 32: Change in burst incidence in response to an isometric handgrip between pregnant and non-pregnant women. df, degrees of freedom; IV, inverse variance; TM1, trimester 1; TM2; TM3, trimester 3.


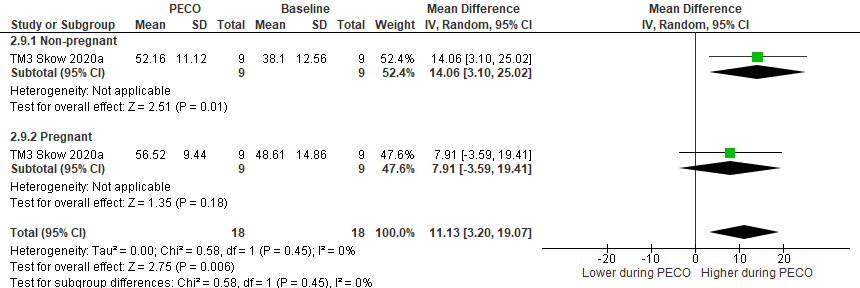


Online Supplement Figure 33: Change in burst incidence in response to a post-exercise circulatory occlusion between pregnant and non-pregnant women. df, degrees of freedom; IV, inverse variance; TM1, trimester 1; TM2; TM3, trimester 3.


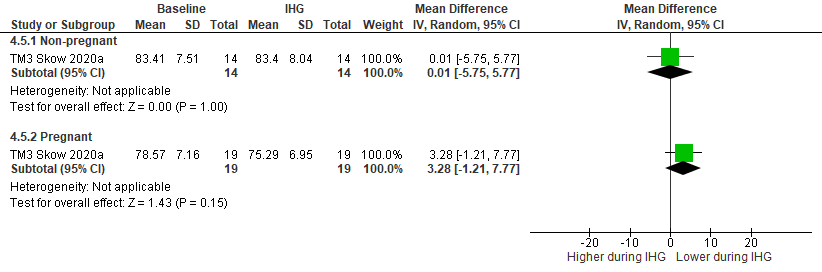


Online Supplement Figure 34: Change in mean arterial pressure in response to an isometric handgrip between pregnant and non-pregnant women. df, degrees of freedom; IV, inverse variance; TM1, trimester 1; TM2; TM3, trimester 3.


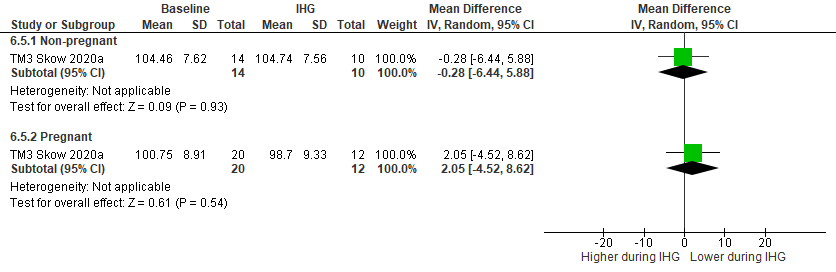


Online Supplement Figure 35: Change in systolic blood pressure in response to an isometric handgrip between pregnant and non-pregnant women. df, degrees of freedom; IV, inverse variance; TM1, trimester 1; TM2; TM3, trimester 3.


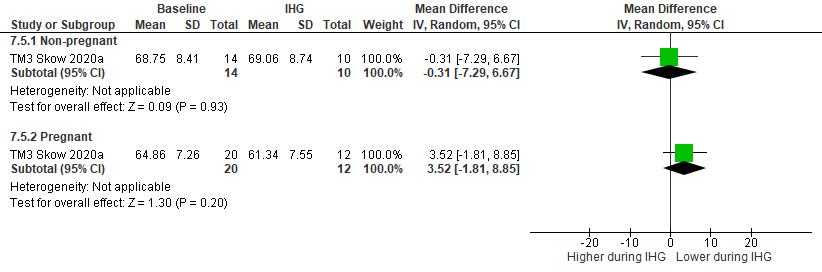


Online Supplement Figure 36: Change in diastolic blood pressure in response to an isometric handgrip between pregnant and non-pregnant women. df, degrees of freedom; IV, inverse variance; TM1, trimester 1; TM2; TM3, trimester 3.


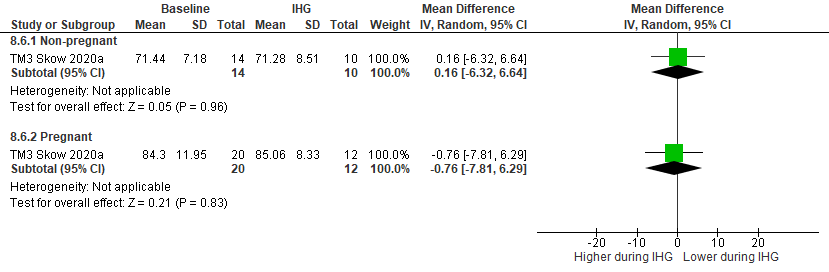


Online Supplement Figure 37: Change in heart rate in response to an isometric handgrip between pregnant and non-pregnant women. df, degrees of freedom; IV, inverse variance; TM1, trimester 1; TM2; TM3, trimester 3.


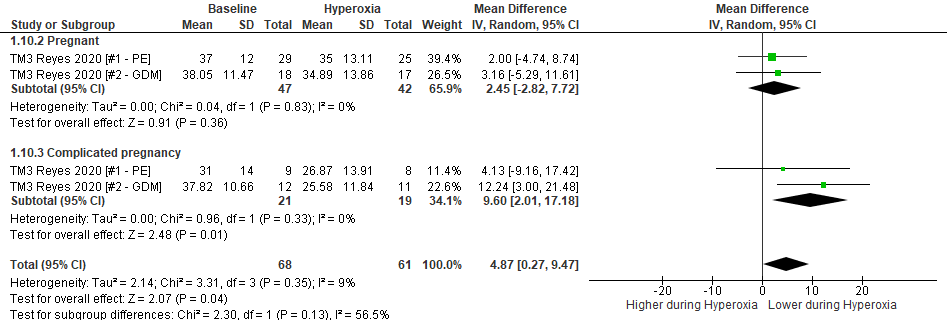


Online Supplement Figure 38: Change in burst frequency in response to hyperoxia between pregnant and non-pregnant women. df, degrees of freedom; IV, inverse variance; TM1, trimester 1; TM2; TM3, trimester 3.


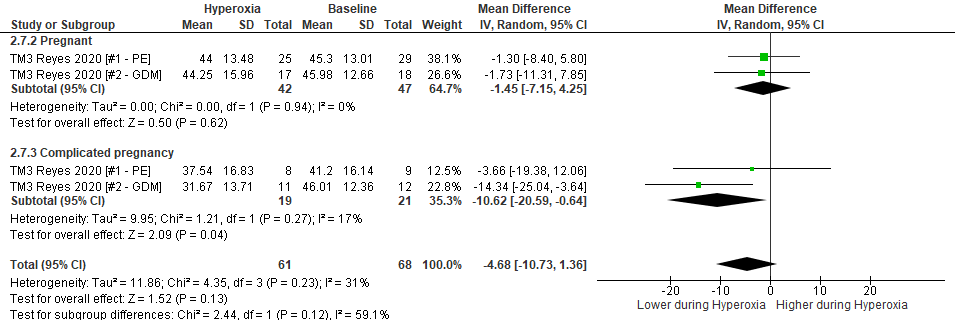


Online Supplement Figure 39: Change in burst incidence in response to hyperoxia between pregnant and non-pregnant women. df, degrees of freedom; IV, inverse variance; TM1, trimester 1; TM2; TM3, trimester 3.


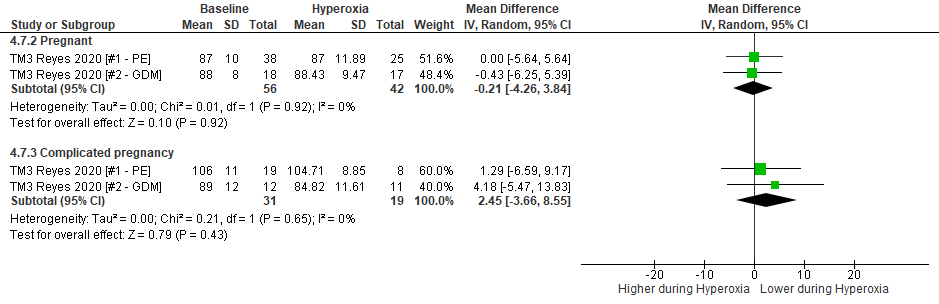


Online Supplement Figure 40: Change in mean arterial pressure in response to hyperoxia between pregnant and non-pregnant women. df, degrees of freedom; IV, inverse variance; TM1, trimester 1; TM2; TM3, trimester 3.


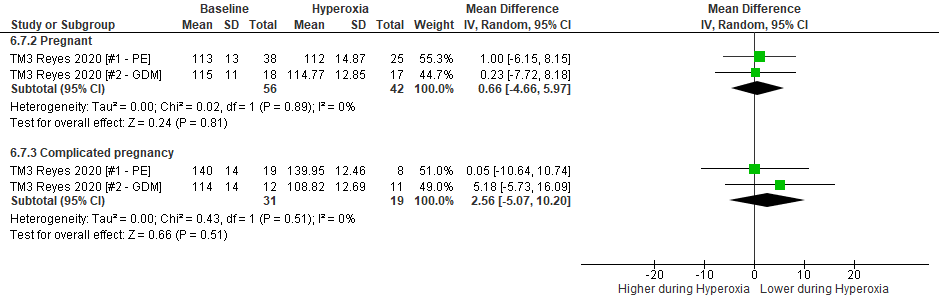


Online Supplement Figure 41: Change in systolic blood pressure in response to hyperoxia between pregnant and non-pregnant women. df, degrees of freedom; IV, inverse variance; TM1, trimester 1; TM2; TM3, trimester 3.


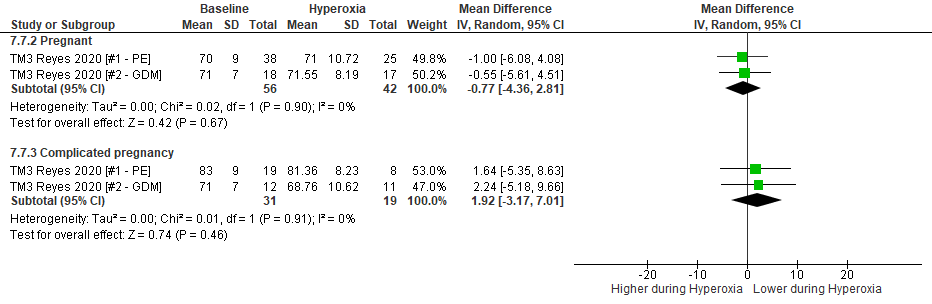


Online Supplement Figure 42: Change in diastolic blood pressure in response to hyperoxia between pregnant and non-pregnant women. df, degrees of freedom; IV, inverse variance; TM1, trimester 1; TM2; TM3, trimester 3.


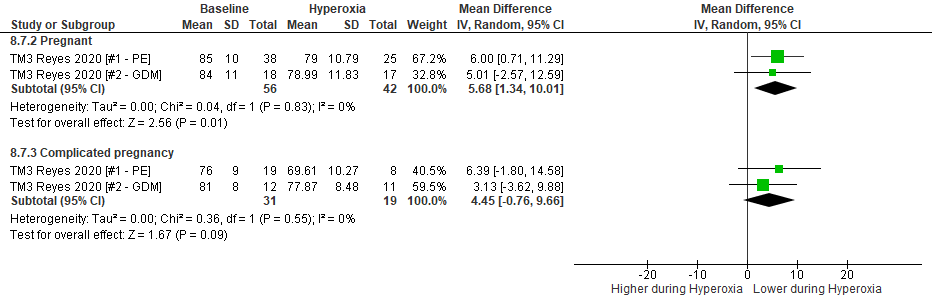


Online Supplement Figure 43: Change in heart rate in response to hyperoxia between pregnant and non-pregnant women. df, degrees of freedom; IV, inverse variance; TM1, trimester 1; TM2; TM3, trimester 3.

1. sympathetic activity (microneurography) AND pregnancy

MEDLINE

SEARCH A
1     (Microneurogra* or micro neurogra* or micro-neurogra*).ti,ab,kf.
2     (muscle sympathetic or muscular sympathetic or msna or muscle sna or muscular sna).mp.
3     (sympathetic nerve activity or sna).ti,ab,kf. or exp Sympathetic Nervous System/
4     exp muscles/ or muscle*.ti,ab,kf.
5     3 and 4
6     2 or 5
7     1 and 6

8 Pregnancy/ or Preconception care/ or pregnan*.ti,hw,kf. or exp Pregnancy Trimesters/ or Peripartum Period/ or Postpartum Period/ or (preconception or pre-conception or pre conception or prepregnancy or pre-pregnancy or pre pregnancy or antenatal or prenatal or perinatal or postnatal or prepartum or antepartum or postpartum or pre partum or ante partum or post partum or puerper* or pregravid* or primigravid* or primiparous or multiparous or nulliparous or multigravid* or trimester* or obstetric*).ti,kf.

SEARCH B:

1     (Microneurogra* or micro neurogra* or micro-neurogra*).ti,ab,kf.
2     (muscle sympathetic or muscular sympathetic or msna or muscle sna or muscular sna).ti,ab,kf.
3     (sympathetic nerve activity or sna).ti,ab,kf. or exp Sympathetic Nervous System/
4     exp muscles/ or muscl*.ti,ab,kf.
5     3 and 4
6     1 or 2 or 5
7     Pregnancy/ or Preconception care/ or pregnan*.ti,hw,kf. or exp Pregnancy Trimesters/ or Peripartum Period/ or Postpartum Period/ or (preconception or pre-conception or pre conception or prepregnancy or pre-pregnancy or pre pregnancy or antenatal or prenatal or perinatal or postnatal or prepartum or antepartum or postpartum or pre partum or ante partum or post partum or puerper* or pregravid* or primigravid* or primiparous or multiparous or nulliparous or multigravid* or trimester* or obstetric*).ti,kf.

8    6 and 7

9    limit 8 to dt=20200929-20220228

EMBASE (same search as MEDLINE)

limit 8 to dd=20200929-20220228

SPORT DISCUS

SEARCH A

1    TI ( Microneurogra* or micro neurogra* or micro-neurogra* ) OR AB ( Microneurogra* or micro neurogra* or micro-neurogra* ) OR KW ( Microneurogra* or micro neurogra* or micro-neurogra* ) OR SU ( Microneurogra* or micro neurogra* or micro-neurogra* ) = 118

2    muscle sympathetic or muscular sympathetic or msna or muscle sna or muscular sna = 763

3    TI ( sympathetic nerve activity or sna ) OR AB ( sympathetic nerve activity or sna ) OR KW ( sympathetic nerve activity or sna ) OR SU sympathetic nervous system = 1513

4    TI ( muscles or muscle* ) OR AB ( muscles or muscle* ) OR KW ( muscles or muscle* ) OR SU ( muscles or muscle* ) = 97930

5    3 and 4 = 407

6    1 OR 2 or 5 = 763

7    MW pregnan* OR TI ( pregnan* or antenatal or prenatal or perinatal or postnatal or prepartum or antepartum or postpartum or pre partum or ante partum or post partum or puerper* or primigravid* or primiparous or multiparous or nulliparous or multigravid* or trimester* or obstetric* ) OR MW ( pregnan* or antenatal or prenatal or perinatal or postnatal or prepartum or antepartum or postpartum or pre partum or ante partum or post partum or puerper* or primigravid* or primiparous or multiparous or nulliparous or multigravid* or trimester* or obstetric* )

8    MH Pregnancy or Pregnancy Complications+ or Pregnancy Outcome or Pregnancy Trimesters+ or Peripartum Period or Postpartum Period

9    7 or 8

10    9 and 6 = 97

SEARCH B:

1    TI ( Microneurogra* or micro neurogra* or micro-neurogra* ) OR AB ( Microneurogra* or micro neurogra* or micro-neurogra* ) OR KW ( Microneurogra* or micro neurogra* or micro-neurogra* ) OR SU ( Microneurogra* or micro neurogra* or micro-neurogra* ) = 118

2    muscle sympathetic or muscular sympathetic or msna or muscle sna or muscular sna = 763

3    TI ( sympathetic nerve activity or sna ) OR AB ( sympathetic nerve activity or sna ) OR KW ( sympathetic nerve activity or sna ) OR SU sympathetic nervous system = 1513

4    TI ( muscles or muscle* ) OR AB ( muscles or muscle* ) OR KW ( muscles or muscle* ) OR SU ( muscles or muscle* ) = 97930

5    3 and 4 = 407

6    1 OR 2 or 5 = 763

7    MW pregnan* OR TI ( pregnan* or antenatal or prenatal or perinatal or postnatal or prepartum or antepartum or postpartum or pre partum or ante partum or post partum or puerper* or primigravid* or primiparous or multiparous or nulliparous or multigravid* or trimester* or obstetric* ) OR MW ( pregnan* or antenatal or prenatal or perinatal or postnatal or prepartum or antepartum or postpartum or pre partum or ante partum or post partum or puerper* or primigravid* or primiparous or multiparous or nulliparous or multigravid* or trimester* or obstetric* )

8    MH Pregnancy or Pregnancy Complications+ or Pregnancy Outcome or Pregnancy Trimesters+ or Peripartum Period or Postpartum Period

9    7 or 8

10    9 and 6 = 97

CINAHL (same search as SPORT DISCUS)

SCOPUS

SEARCH A

1. TITLE-ABS-KEY ( microneurogra*  OR  micro  AND neurogra*  OR  micro-neurogra* )
2. TITLE-ABS-KEY ( muscles  OR  muscle* )
3. ( TITLE-ABS-KEY ( muscle  AND  sympathetic )  OR  TITLE-ABS-KEY ( muscular  AND  sna )  OR  TITLE-ABS-KEY ( muscular  AND  sympathetic )  OR  TITLE-ABS-KEY ( msna )  OR  TITLE-ABS-KEY ( muscle  AND  sna ) )
4. ( TITLE-ABS-KEY ( sympathetic  AND  nerve  AND  activity )  OR  TITLE-ABS-KEY ( sna )  OR  TITLE-ABS-KEY ( "sympathetic nervous system" ) )

3 and 4

2 or 5

1 and 6

SEARCH B

1 TITLE-ABS-KEY ( microneurogra*  OR  micro  AND neurogra*  OR  micro-neurogra* )

2 ( TITLE-ABS-KEY ( muscle  AND  sympathetic )  OR  TITLE-ABS-KEY ( muscular  AND  sna )  OR  TITLE-ABS-KEY ( muscular  AND  sympathetic )  OR  TITLE-ABS-KEY ( msna )  OR  TITLE-ABS-KEY ( muscle  AND  sna ) )

3 ( TITLE-ABS-KEY ( sympathetic  AND  nerve  AND  activity )  OR  TITLE-ABS-KEY ( sna )  OR  TITLE-ABS-KEY ( "sympathetic nervous system" ) )

4  3 and 4

5  1 or 2 or 5

6  pregnan* or antenatal or prenatal or perinatal or postnatal or prepartum or antepartum or postpartum or “pre partum” or “ante partum” or “post partum” or puerper* or primigravid* or primiparous or multiparous or nulliparous or multigravid* or trimester* or obstetric*

7   6 and 7

Web of Science

SEARCH B

1    microneurogra* OR "micro neurogra*" OR micro-neurogra* (Topic)

2    "sympathetic nerve activity" OR sna OR "sympathetic nervous system" (Topic) and "muscle sympathetic" or "muscular sna" or "muscular sympathetic" or msna or "muscle sna" (Topic)

3    "muscle sympathetic" OR "muscular sna" (Topic)

4    pregnan* or antenatal or prenatal or perinatal or postnatal or prepartum or antepartum or postpartum or “pre partum” or “ante partum” or “post partum” or puerper* or primigravid* or primiparous or multiparous or nulliparous or multigravid* or trimester* or obstetric* (Topic)

5    1 or 2 or 3

6    5 and 4

COCHRANE

SEARCH B

#1    MeSH descriptor: [Pregnancy] explode all trees

#2    MeSH descriptor: [Pregnancy Trimesters] explode all trees

#3    MeSH descriptor: [Peripartum Period] explode all trees

#4    MeSH descriptor: [Postpartum Period] explode all trees

#5    preconception or pre-conception or pre conception or prepregnancy or pre-pregnancy or pre pregnancy or antenatal or prenatal or perinatal or postnatal or prepartum or antepartum or postpartum or pre partum or ante partum or post partum or puerper* or pregravid* or primigravid* or primiparous or multiparous or nulliparous or multigravid* or trimester* or obstetric*

#6    #1 or #2 or #3 or #4 or #5

#7    MeSH descriptor: [Sympathetic Nervous System] explode all trees

#8    Microneurogra* or micro neurogra* or micro-neurogra*

#9    muscle sympathetic or muscular sympathetic or msna or muscle sna or muscular sna

#10    MeSH descriptor: [Muscles] explode all trees

#11    muscl*

#12    #10 or #11

#13    #12 and #7

#14    #13 or #8 or #9

#15    #14 and #6
